# Supplementary material for: Conserved mammalian modularity of quantitative trait loci revealed human functional orthologs in blood pressure control
Source: PLoS One. 2020 Jul 23;15(7):e0235756. doi: 10.1371/journal.pone.0235756 (PMC7377405; doi:10.1371/journal.pone.0235756)
Supplement: S1 Table — (DOCX) [file pone.0235756.s002.docx]

**Supplemental Table 1: Rat QTLs and genes residing in the intervals harboring them that correspond to human GWAS genes trapped by congenic knock in genetics**.

| **RatQTL** | **Rat Gene** | **Rat Chr spans** | **Human GWAS gene**  **(SNPs, positions)** | **Human CHR** |
| --- | --- | --- | --- | --- |
| ***C7QTL1*** | *Cand1* | 62113404..62151493 | *CAND1* | 12 |
|  | *Llph* | 65223409..65228370 | *LLPH* | 12 |
|  | *Arhgef25* | 70646945..70654051 | *ARHGEF25* | 12 |
|  | *Nxph4* | 71006677..71014869 | (No GWAS gene) | 12 |
|  | *Rdh16(Rdh2)* | 71151962..71152405 | (No GWAS gene) | 12 |
|  | *Tac3(Tac2)* | 71196136..71203264 | (No GWAS gene) | 12 |
|  | *Snx31* | 75456983..75512331 | *SNX31* | 8 |
|  | *Ncald* | 76320073..76747866 | *NCALD* | 8 |
|  | *Azin1* | 77448669..77475344 | *AZIN1* | 8 |
|  | *Trhr* | 83121134..83161215 | *TRHR* | 8 |
|  | *Trps1* | 90113508..90343325 | *TRPS1* | 8 |
|  | *Nov* | 95015159..95022179 | *NOV* | 3 |
|  | *Zfat* | 108980615..109149677 | *ZFAT* | 8 |
|  | *Trappc9* | 114185706..114244503 | *TRAPPC9* | 8 |
|  | *Ptk2* | 114371979..114525553 | *PTK2* | 8 |
|  | *Gpr20* | 114865346..114875413 | *GPR20* | 8 |
|  | *Cyp11b2* | 116151128..116157542 | *CYP11B2* | 8 |
|  | *Plec* | 117215919..117277093 | *PLEC* | 8 |
|  | *Nol12* | 120152929..120158543 | *NOL12* | 22 |
|  | ***Triobp*** | 120168221..120228826  1.7 Mb | ***TRIOBP*** (human exon spans 37696988..37776556)  Intron3 rs4820296, (at 37708698)  Exon7 rs12628603, (at 37723206)  3’UTR rs1129448, (at 37774019) | 22 |
|  | ***Tnrc6b*** | 121922421..122139717 | ***TNRC6B*** (human exon spans 40044817..40335808)  3’UTR rs470113, (at 40333610) | 22 |
|  | *Xrcc6* | 123244581..123265278 | *XRCC6* | 22 |
|  | *Brd1* | 129366020..129413531 | *BRD1* | 22 |
|  | *Plxnb2* | 129821099..129847380 | *PLXNB2* | 22 |
| ***C7QTL2*** | *Atp2b1* | 41153926..41262537 | *ATP2B1* | 12 |
|  | *Syt1* | 50097034..50289950 | *SYT1* | 12 |
|  | *Rab3ip/Cnot2* | 59932332..59976359 | *RAB3IP/CNOT2* | 12 |
| ***C8QTL1*** | *Nrg4* | 58364273..58430812 | *NRG4* | 15 |
|  | ***Ulk3*** | 62146130..62152150  4.9 kb | ***ULK3*** (human exon spans 74836166..74843211)  **intergenic rs6495122**, (at 74833304) | 15 |
|  | *CPLX3* | 62157074..62163836  85 kb | *CPLX3* (human exon spans 74826627-74831797) | 15 |
|  | *Cyp1a1* | 62249046..62255081  27 kb | *CYP1A1* (human exon spans 74719542-74725528)  23 kb | 15 |
|  | ***Cyp1a2*** | 62228271-62235203  52 kb | ***CYP1A2*** (human exon spans 74748845-74756607)  28 kb | 15 |
|  |  |  | **intergenic rs1378942**, (at 74785026)  3.5 kb | 15 |
|  | *Csk* | 62183286-62187910  23.5 kb | *CSK* (human exon spans 74788544-74803197)  (No GWAS SNP)  9.6 kb | 15 |
|  | *Lman1L* | 62164378-62177220  12.8 kb | *LMAN1L* (human exon spans74812835-74825754)  (No GWAS SNP)  423 kb | 15 |
|  | ***Ccdc33*** | 62586934..62686180 | ***CCDC33*** (human exon spans 74236289..74336141)  Intron2 rs351157, (at 74259690)  Intron3 rs4887123, (at 74265653)  Intron3 rs94899, (at 74266268) | 15 |
|  | *Thsd4* | 64569306..65166353 | *THSD4* | 15 |
|  | *Cln6* | 67461962..67476917 | *CLN6* | 15 |
|  | *Calml4* | 67482026..67494105 | *CALML4* | 15 |
|  | *Pias1* | 67500467..67597762 | *PIAS1* | 15 |
|  | *Smad3* | 68290927..68397727 | *SMAD3* | 15 |
|  | *Plekho2* | 70781037..70804900 | *PLEKHO2* | 15 |
|  | *Myo1e* | 76241676..76432611 | *MYO1E* | 15 |
|  | *C2cd4b* | 77922752..77924242 | *C2CD4B* | 15 |
|  | *Mlip* | 83981876..84206700 | *MLIP* | 6 |
|  | *Phip* | 89916080..90014067 | *PHIP* | 6 |
|  | *Bckdhb* | 90982516..91173706 | *BCKDHB* | 6 |
|  | *Tbx18* | 94860116..94888125 | *TBX18* | 6 |
| ***C8QTL2*** | *Yap1* | 6131653..6202586 | *YAP1* | 11 |
|  | *Pgr* | 7113895..7172761 | *PGR* | 11 |
|  | *Sesn3* | 12768558..12824247 | *SESN3* | 11 |
|  | ***Mtnr1b*** | 14446990..14461614 | ***MTNR1B*** (human exon spans 92969623..92982782)  Intron1 rs10830963, (at 92975544) | 11 |
|  | *Mrpl4* | 22078081..22084096 | *MRPL4* | 19 |
|  | *Rgl3* | 23035212..23055282 | *RGL3* | 19 |
|  | *Elavl3* | 23084379..23118191 | *ELAVL3* | 19 |
|  | *Tbx20* | 25880011..25934699 | *TBX20* | 7 |
|  | *Eepd1* | 26672254..26779328 | *EEPD1* | 7 |
|  | ***Snx19*** | 31526141..31563756 | ***SNX19*** (human exon spans 130877871..130916487)  Exon1 rs1050081, (at 130914752)  Exon6 rs2276098, (at 130907991)  Intron9 rs948086, (at 130883174) | 11 |
|  | *Adamts8* | 32044601..32064092 | *ADAMTS8* | 11 |

Footnote for table: Fig. 1 of the text defines the chromosome regions containing QTLs and the gene residing in the intervals containing these QTLs. The rat and human genome regions are adopted from UCSC Rat Rno5.0 and UCSC Human GRCh38.p12 respectively (<https://rgd.mcw.edu/rgdweb/search/genes.html>). Human GWAS genes are all from (*Nat Genet* 2018;50:1412). Only the genes underlined and in bold were analyzed further in the current work, because they are functional candidate genes for the human QTLs (Table 1 of text). CHR, Chromosome. The human GWAS genes with corresponding rat QTL candidates are indicated by bold underlined letters. *C7QTL1*-residing region contains 783 genes (569 protein-coding genes, 108 pseudo, 104 non-coding RNAs, 1 tRNA); *C7QTL2*-residing region contains 161 genes (95 protein-coding genes, 38 pseudo genes, 28 non-coding RNAs); *C8QTL1*-residing regions contains 464 genes (331 protein-coding genes, 71 pseudo genes, 60 non-coding RNAs); *C8QTL2*-residing region contains 161 genes (253 protein-coding genes, 106 pseudo genes, 26 non-coding RNAs).
