# Supplementary material for: Conserved mammalian modularity of quantitative trait loci revealed human functional orthologs in blood pressure control
Source: PLoS One. 2020 Jul 23;15(7):e0235756. doi: 10.1371/journal.pone.0235756 (PMC7377405; doi:10.1371/journal.pone.0235756)
Supplement: S2 Table — (DOCX) [file pone.0235756.s003.docx]

**Supplemental Table 2: A survey of sequence homologies between humans and the rat for non-coding GWAS SNPs marking the positions of human genes corresponding to the rat QTLs for blood pressure**

| **Human SNP/**  **Marked gene** | Homology | Note |
| --- | --- | --- |
| rs4820296*/* *TRIOBP*  (intron3) | No | Haphazard hits in 1.1Kb sequence used for blast; several mini- regions of 31-34 bp of homology randomly distributed on rat Chr7, but not in the right region. |
| rs12628603*/* *TRIOBP*  (exon7) | No | No hits in 1Kb sequence used for blast on Chr7 |
| rs1129448*/* *TRIOBP*  (3’UTR) | Yes | 229 bp region hit with 88.1 % homology containing the SNP region |
| rs470113*/* *TNRC6B*  (3’UTR) | No | No hits in 1Kb sequence used for blast on Chr7  (22 bp hit not at the right region on Chr7) |
| Rs17249754/*ATP2B1*  (intergenic) | No | No hits in 1Kb sequence used for blast on Chr7 |
| rs1401982/*ATP2B1*  (intron18) | No | No hits in 1Kb sequence used for blast on Chr7  (21 bp hit not at the right region on Chr7) |
| rs6495122*/* *ULK3*  (intergenic) | No | Haphazard hits in 1 Kb sequence used for blast; several mini- regions of 26-171 bp of homology randomly distributed on rat Chr8, but not in the right region. |
| rs1378942/CYP1A2/CSK  (intergenic) | No | No hits in 1Kb sequence used for blast on Chr8  (56 bp hit not at the right region on Chr8) |
| rs351157*/* *CCDC33*  (intron2) | No | No hits in 1Kb sequence used for blast on Chr8  (78 bp hit not at the right region on Chr8) |
| rs4887123*/* *CCDC33*  (intron3) | No | Haphazard hits in 1 Kb sequence used for blast; several mini- regions of 31-36 bp of homology randomly distributed on rat Chr8, but not in the right region. |
| *rs94899/* *CCDC33*  (intron3) | No | Haphazard hits in 1 Kb sequence used for blast; several mini- regions of 31-39 bp of homology randomly distributed on rat Chr8, but not in the right region. |
| rs10830963/ *MTNR1B*  (intron) | No | No hits in 1Kb sequence used for blast on Chr8 |
| rs1050081/*SNX19*  (exon1) | No | The SNP is absent but a surrounding region of 614 bp (total 1Kb) is very homologue (88%) |
| rs2276098/*SNX19*  (exon6) | No | The SNP is absent but a surrounding of 283bp (total 1Kb) is very homologue (87%) |
| rs948086/*SNX19*  (intron9) | No | No hits in 1Kb sequence used for blast on Chr8 |

Footnote: Gene names are given in legends of Table 1 in the text. Appropriate sequences surrounding a SNP in question was blasted into the rat genome (RGSC 5.0/rn5) at:

<https://genome.ucsc.edu/cgi-bin/hgGateway>. Entries are:

>gnl|dbSNP|rs4820296|allelePos=1000|totalLen=1100|taxid=9606|snpclass=1|alleles='A/T'|mol=Genomic|build=151

>gnl|dbSNP|rs12628603|allelePos=501|totalLen=1001|taxid=9606|snpclass=1|alleles='A/C/G'|mol=Genomic|build=151

>gnl|dbSNP|rs1129448|allelePos=501|totalLen=1001|taxid=9606|snpclass=1|alleles='A/C/G/T'|mol=Genomic|build=151

>gnl|dbSNP|rs470113|allelePos=501|totalLen=1001|taxid=9606|snpclass=1|alleles='A/G'|mol=Genomic|build=151

>gnl|dbSNP|rs1401982|allelePos=501|totalLen=1001|taxid=9606|snpclass=1|alleles='C/T'|mol=Genomic|build=151

>gnl|dbSNP|rs17249754|allelePos=501|totalLen=1001|taxid=9606|snpclass=1|alleles='A/G'|mol=Genomic|build=151

>gnl|dbSNP|rs6495122|allelePos=501|totalLen=1001|taxid=9606|snpclass=1|alleles='A/C'|mol=Genomic|build=151

>gnl|dbSNP|rs1378942|allelePos=501|totalLen=1001|taxid=9606|snpclass=1|alleles='A/G/T'|mol=Genomic|build=151

>gnl|dbSNP|rs351157|allelePos=501|totalLen=1001|taxid=9606|snpclass=1|alleles='A/C'|mol=Genomic|build=151

>gnl|dbSNP|rs4887123|allelePos=501|totalLen=1001|taxid=9606|snpclass=1|alleles='C/G'|mol=Genomic|build=151

>gnl|dbSNP|rs94899|allelePos=501|totalLen=1001|taxid=9606|snpclass=1|alleles='C/T'|mol=Genomic|build=151

>gnl|dbSNP|rs10830963|allelePos=501|totalLen=1001|taxid=9606|snpclass=1|alleles='C/G'|mol=Genomic|build=151

>gnl|dbSNP|rs1050081|allelePos=501|totalLen=1001|taxid=9606|snpclass=1|alleles='C/G'|mol=Genomic|build=151

>gnl|dbSNP|rs2276098|allelePos=501|totalLen=1001|taxid=9606|snpclass=1|alleles='A/G'|mol=Genomic|build=151

>gnl|dbSNP|rs948086|allelePos=501|totalLen=1001|taxid=9606|snpclass=1|alleles='A/G'|mol=Genomic|build=151
