# Supplementary material for: Conserved mammalian modularity of quantitative trait loci revealed human functional orthologs in blood pressure control
Source: PLoS One. 2020 Jul 23;15(7):e0235756. doi: 10.1371/journal.pone.0235756 (PMC7377405; doi:10.1371/journal.pone.0235756)
Supplement: S3 Table — (DOCX) [file pone.0235756.s004.docx]

**Supplemental Table 3. Survey of non-coding GWAS SNP conservations/homology during primate evolution**

Recent ancient (Arnold et al. 2010)

| **Human SNP/**  **Marked gene** | **Chimpanzee** | **Bonobo** | **Gorilla** | **Orangu**  **tan** | **Gibbons** | **Old World monkeys** | **New World monkeys** |
| --- | --- | --- | --- | --- | --- | --- | --- |
| *rs4820296/* *TRIOBP*  (intron3) | homology | homology | homology | homology | homology | homology | No homology |
| *rs12628603/* *TRIOBP*  (exon7) | homology | homology | homology | homology | homology | homology | homology but not at the right chr |
| *rs1129448/* *TRIOBP*  (3’UTR) | homology | homology | homology | homology | homology | homology | No homology |
| *rs470113/* *TNRC6B*  (3’UTR) | homology | homology | homology | homology | homology | homology | homology/Squirrel Monkey  No homology/ Marmoset, SNP absent but surrounding sequence conserved |
| *rs6495122/* *ULK3*  (intergenic) | homology | homology | homology | homology | homology | homology | No homology, SNP absent but surrounding sequence conserved |
| rs1378942/CYP1A2  (intergenic) | homology | homology | homology | homology | homology | homology | homology |
| *rs351157/* *CCDC33*  (intron2) | homology | homology | homology | homology | homology | No homology, SNP absent but surrounding sequence conserved | homology |
| *rs4887123/* *CCDC33*  (intron3) | homology | homology | homology | homology | homology | homology | homology |
| *rs94899/* *CCDC33*  (intron3) | homology | homology | homology | homology | homology | homology | homology |
| rs10830963/ *MTNR1B*  (intron1) | homology | homology | homology | homology | homology | homology | No homology |
| rs1050081/*SNX19*  (exon1) | homology | homology | homology | homology | homology | homology | homology |
| rs2276098/*SNX19*  (exon6) | homology | homology | homology | homology | homology | homology | homology |
| rs948086/*SNX19*  (intron9) | homology | homology | homology | homology | homology | homology | No homology/ SNP absent but surrounding sequence homologue |

Footnote: Gene names are given in the legend for Table 1 in the text. Homology indicates that the SNP and/or surrounding sequences are conserved. Old World monkeys are represented by Rhesus macaque and baboon; New World monkeys are represented by marmoset and squirrel monkey. Searches were done at <https://genome.ucsc.edu/cgi-bin/hgGateway>. Entries are:

>gnl|dbSNP|rs4820296|allelePos=1000|totalLen=1100|taxid=9606|snpclass=1|alleles='A/T'|mol=Genomic|build=151

>gnl|dbSNP|rs12628603|allelePos=501|totalLen=1001|taxid=9606|snpclass=1|alleles='A/C/G'|mol=Genomic|build=151

>gnl|dbSNP|rs1129448|allelePos=501|totalLen=1001|taxid=9606|snpclass=1|alleles='A/C/G/T'|mol=Genomic|build=151

>gnl|dbSNP|rs470113|allelePos=501|totalLen=1001|taxid=9606|snpclass=1|alleles='A/G'|mol=Genomic|build=151

>gnl|dbSNP|rs6495122|allelePos=501|totalLen=1001|taxid=9606|snpclass=1|alleles='A/C'|mol=Genomic|build=151

>gnl|dbSNP|rs1378942|allelePos=501|totalLen=1001|taxid=9606|snpclass=1|alleles='A/G/T'|mol=Genomic|build=151

>gnl|dbSNP|rs351157|allelePos=501|totalLen=1001|taxid=9606|snpclass=1|alleles='A/C'|mol=Genomic|build=151

>gnl|dbSNP|rs4887123|allelePos=501|totalLen=1001|taxid=9606|snpclass=1|alleles='C/G'|mol=Genomic|build=151

>gnl|dbSNP|rs94899|allelePos=501|totalLen=1001|taxid=9606|snpclass=1|alleles='C/T'|mol=Genomic|build=151

>gnl|dbSNP|rs10830963|allelePos=501|totalLen=1001|taxid=9606|snpclass=1|alleles='C/G'|mol=Genomic|build=151

>gnl|dbSNP|rs1050081|allelePos=501|totalLen=1001|taxid=9606|snpclass=1|alleles='C/G'|mol=Genomic|build=151

>gnl|dbSNP|rs2276098|allelePos=501|totalLen=1001|taxid=9606|snpclass=1|alleles='A/G'|mol=Genomic|build=151

>gnl|dbSNP|rs948086|allelePos=501|totalLen=1001|taxid=9606|snpclass=1|alleles='A/G'|mol=Genomic|build=151
