# Supplementary material for: Conserved mammalian modularity of quantitative trait loci revealed human functional orthologs in blood pressure control
Source: PLoS One. 2020 Jul 23;15(7):e0235756. doi: 10.1371/journal.pone.0235756 (PMC7377405; doi:10.1371/journal.pone.0235756)
Supplement: S4 Table — (DOCX) [file pone.0235756.s005.docx]

**Supplemental Table 4 (A). Amino acid alignment and missense mutations in codons for neurexophilin 4 (NXPHX) in humans and rats**

Human_NXPH4 MRLLPEWFLLLFGPWLLRKAVSAQIPESGRPQYLGLRPAAAGAGAPGQQLPEPRSSDGLG 60

DSS_Rat_Nxph4 MRLLPEWLLLLFGPWLLRKVISGQIVESGRPQYLDLRPAMAGGGARGQQLPVPASSEGLN 60

*******:***********.:*.** ********.**** **.** ***** * **:**.

Human_NXPH4 VGRAWSWAWPTNHTGALARAGAAGALPAQRTKRKPSIKAARAKKIFGWGDFYFRVHTLKF 120

DSS_Rat_Nxph4 PVRSWSWAWPANHTGALARPGAAGGPPVPRTKRKPSIKAARAKKIFGWGDFYFRVHTLKF 120

*:******:******** ****. *. *******************************

Human_NXPH4 SLLVTGKIVDHVNGTFSVYFRHNSSSLGNLSVSIVPPSKRVEFGGVWLPGPVPHPLQSTL 180

DSS_Rat_Nxph4 SLLVTGKIVDHVNGTFSVYFRHNSSSLGNLSVSIVPPSKRVEFGGVWLPGPAPHPLQSTL 180

***************************************************.********

Human_NXPH4 ALEGVLPGLGPPLGMAAAAAGPGLGGSLGGALAGPLGGALGVPGAKESRAFNCHVEYEKT 240

DSS_Rat_Nxph4 ALEGVLPGLGPPLGMA----GQGLGGNLGGALAGPLGGALGVPGAKESRAFNCHVEYEKT 236

********** ***** * ****.*********************************

Human_NXPH4 NRARKHRPCLYDPSQVCFTEHTQSQAAWLCAKPFKVICIFVSFLSFDYKLVQKVCPDYNF 300

DSS_Rat_Nxph4 NRARKHRPCLYDPSQVCFTEHTQSQAAWLCAKPFKVICIFVSFLSFDYKLVQKVCPDYNF 296

************************************************************

Human_NXPH4 QSEHPYFG 308

DSS_Rat_Nxph4 QSEHPYFG 304

********

Footnote for table: * indicates amino acid identity (92%) between human and the rat. Probable human missense mutations (The Genomes Project et al. 2015) are shaded, which were curated from <https://www.ncbi.nlm.nih.gov/snp/?term=nxph4+missense> and as of July 9, 2019. Only those missense mutations with minor alleles that were observed at least 2 times (marked in red) in the tested populations are included with the validation status by 1000Genomes. Amino acids in blue indicate that the minor allele has been observed more than 10 times. The rat missense mutation has been experimentally confirmed and shaded in green. DSS, Dahl salt-sensitive rats.

**Supplemental Table 4 (B). Amino acid alignment and missense mutations in codons for retinol dehydrogenase 16 (RDH16) in humans and rats**

Human_RDH16 MWLYLAVFVGLYYLLHWYRERQVLSHLRDKYVFITGCDSGFGKLLARQLDARGLRVLAAC 60

DSS_Rat_Rdh16 MWLYLLALVGLWNLLRLFRERKVVSHLQDKYVFITGCDSGFGNLLARQLDRRGMRVLAAC 60

***** .:***: **: :***:*:***:**************:******* **:******

Human_RDH16 LTEKGAEQLRGQTSDRLETVTLDVTKTESVAAAAQWVKECVRDKGLWGLVNNAGISLPTA 120

DSS_Rat_Rdh16 LTEKGAEQLRSKTSDRLETVILDVTKTESIVAATQWVKERVGNTGLWGLVNNAGISGHLG 120

**********.:******** ********:.**:***** * :.************ .

Human_RDH16 PNELLTKQDFVTILDVNLLGVIDVTLSLLPLVRRARGRVVNVSSVMGRVSLFGGGYCISK 180

DSS_Rat_Rdh16 PNEWMNKQNIASVLDVNLLGMIEVTLSTVPLVRKARGRVVNVASIAGRLSFCGGGYCISK 180

*** :.**::.::*******:*:**** :****:********:*: **:*: ********

Human_RDH16 YGVEAFSDSLRRELSYFGVKVAMIEPGYFKTAVTSKERFLKSFLEIWDRSSPEVKEAYGE 240

DSS_Rat_Rdh16 YGVEAFSDSLRRELSYFGVKVAIVEPGFFRTDVTNGVTLSSNFQMLWDQTSSEVREVYGE 240

**********************::***:*:* **. : ..* :**::* **:*.***

Human_RDH16 KFVADYKKSAEQMEQKCTQDLSLVTNCMEHALIACHPRTRYSAGWDAKLLYLPMSYMPTF 300

DSS_Rat_Rdh16 NYLASYLKTLNGLDQRCNKDLSLVTDCMEHALTSCHPRTRYSAGWDAKFFYLPMSYLPTF 300

:::*.* * : ::*:*.:******:****** :**************::******:***

Human_RDH16 LVDAIMYWVSPSPAKAL 317

DSS_Rat_Rdh16 LVDALFYWTSPKPEKAL 317

****::**.**.* ***

Footnote for table: * indicates amino acid identity (77%) between human and the rat. Probable human missense mutations (The Genomes Project et al. 2015) are shaded, which were curated from <https://www.ncbi.nlm.nih.gov/snp/?term=rdh16+missense> and as of July 9, 2019. Only those missense mutations with minor alleles that were observed at least 2 times (marked in red) in the tested populations are included with the validation status by 1000Genomes. Amino acids in blue indicate that the minor allele has been observed more than 10 times. The rat missense mutation has been experimentally confirmed and shaded in green. DSS, Dahl salt-sensitive rats.

**Supplemental Table 4 (C). Amino acid alignment and missense mutations in codons for tachykinin precursor 3 (TAC3) in humans and rats**

Human_TAC3 MRIMLLFTAILAFSLAQSFGAVCKEPQEEVVPGGGRSKRDPDLYQ----LLQRLFKS-HS 55

DSS_Rat_Tac3 MRSAMLFAAVLALSLAWTFGAACEEPQEQ----GGRLSKDSDLSLLPPPLLRRLYDSRSI 56

** :**:*:**:*** :***.*:****: *** .:* ** **:**:.*

Human_TAC3 SLEGLLKALSQASTDPKESTSPEKRDMHDFFVGLMGKRSVQPDSPTDVNQENVPSFGILK 115

DSS_Rat_Tac3 SLEGLLKVLSKASVGPKETSLPQKRDMHDFFVGLMGKRNSQPDTPADVVEENTPSFGVLK 116

*******.**:**..***:: *:***************. ***:*:** :**.****:**

Human_TAC3 YPPRAE 121

DSS_Rat_Tac3 ------ 116

Footnote for table: * indicates amino acid identity (86%) between human and the rat. Probable human missense mutations (The Genomes Project et al. 2015) are shaded, which were curated from <https://www.ncbi.nlm.nih.gov/snp/?term=tac3+missense> and as of July 9, 2019. Only those missense mutations with minor alleles that were observed at least 2 times (marked in red) in the tested populations are included with the validation status by 1000Genomes. Amino acids in blue indicate that the minor allele has been observed more than 10 times. The rat missense mutation has been experimentally confirmed and shaded in green. DSS, Dahl salt-sensitive rats.

**Supplemental Table 4 (D). Amino acid alignment and missense mutations in codons for TRIO and F-actin binding protein (TRIOBP) in humans and rats**

Human_TRIOBP --MEEVPGDALC---EHFEANILTQNRCQNCFHPEEAHGARYQELRSPSGAEVPYCDLPR 55

DSS_Rat_Triobp MSMEQDTRALLPTQGTAWATASVPVARLQ--GPQGDSHQACSQEPHSPPSAEAPYCDLPR 58

**: * : : : * * ::* * ** :** .**.*******

Human_TRIOBP CPPAPEDPLSASTSGCQSVVDPGL--RPGPKRGPSPSAGLPEEGPTAAPRSRSRELEAVP 113

DSS_Rat_Triobp CPPALQDPLHTTTCVGQSVDNLGLGLDQEPQRI-----GSPTTALPADPRNRHRDPEAIP 113

**** :*** ::*. *** : ** *:* * * . * **.* *: **:*

Human_TRIOBP YLEGLTTSLCGSCNEDPGSDPTSSPDSATPDDTSNSSSVDWDTVERQEEEAPSWDELAVM 173

DSS_Rat_Triobp YLEGPAYSTDEHKDEDPNSNTSSSQDSNTPHDTSNSSSVDWDTVERPG-VVPNRNRLTMM 172

**** : * :***.*: :** ** **.*************** .*. :.*::*

Human_TRIOBP IPRRPREGPRADSSQRAPSLLTRSPVGGDAAGQKKEDTGGGGRSAGQHWARLRGESGL-S 232

DSS_Rat_Triobp IPRRPQEGLRTDSAQK----VTRSPARGDTAGQRKENSGSGGQSAGQHWVKLRSESGYFS 228

*****:** *:**:*: :****. **:***:**::*.**:******.:**.*** *

Human_TRIOBP LERHRSTLTQASSMTPHSGPRSTTSQASPAQRDTAQAASTREIPRASSPHRITQRDTSRA 292

DSS_Rat_Triobp LERQRSGQTQASSGTPPSGPRTTTQ-ASSAQRDVSQAASAQEAPQTSSLPRNTQRDT--- 284

***:** ***** ** ****:**. ** ****.:****::* *::** * *****

Human_TRIOBP SSTQQEISRASSTQQETSRASSTQEDTPRASSTQEDTPRASSTQWNTPRASSPSRSTQLD 352

DSS_Rat_Triobp -------------------------------------------QRSTPRTSSPSRVSQRD 301

* .***:***** :* *

Human_TRIOBP NPRTSSTQQDNPQTSFPTCTPQRENPRTPCVQQDDPRASSPNRTTQRENSRTSCAQRDNP 412

DSS_Rat_Triobp TPRIMSTQ---------------------------------------------------- 309

.** ***

Human_TRIOBP KASRTSSPNRATRDNPRTSCAQRDNPRASSPSRATRDNPTTSCAQRDNPRASRTSSPNRA 472

DSS_Rat_Triobp ------------------------------------------------------------ 309

Human_TRIOBP TRDNPRTSCAQRDNPRASSPSRATRDNPTTSCAQRDNPRASRTSSPNRATRDNPRTSCAQ 532

DSS_Rat_Triobp -----------RKNTPLSSPLRPT-------------PETLKTSAP-------------- 331

*.* *** * * *.: :**:*

Human_TRIOBP RDNPRASSPNRAARDNPTTSCAQRDNPRASRTSSPNRATRDNPRTSCAQRDNPRASSPNR 592

DSS_Rat_Triobp EDGPH-------------------------------------------------VTS--- 339

.*.*: .:*

Human_TRIOBP ATRDNPTTSCAQRDNPRASRTSSPNRATRDNPRTSCAQRDNPRASSPNRTTQQDSPRTSC 652

DSS_Rat_Triobp -------PLCAQD-------SSLNRTTQRNSSRTSCAQRNNPRTSSPNRTTQRDNPRTSC 385

*** :* . : **. *******:***:********:*.*****

Human_TRIOBP ARRDDPRASSPNRTIQQENPRTSCALRDNPRASSPSRTIQQENPRTSCAQRDDPRASSPN 712

DSS_Rat_Triobp AQRNNPRTSSPNRTTQQDNPRTSCAQRNNPRTSSPSRTTQHDNPRTSCAQRDNPRTSSPN 445

*:*::**:****** **:******* *:***:****** *::**********:**:****

Human_TRIOBP RTTQQENPRTSCARRDNPRASSRNRTIQRDNPRTSCAQRDNPRASSPNRTIQQENLRTSC 772

DSS_Rat_Triobp RTTQQDNPRMSCAQRNNPRTSSPNRTTQRDNLRTSCAQRNNPRTSSPNRTIHQDNPRTSC 505

*****:*** ***:*:***:** *** **** *******:***:*******:*:* ****

Human_TRIOBP TRQDNPRTSSPNRATRDNPRTSCAQRDNLRASSPIRATQQDNPRTCIQQNIPRSSSTQQD 832

DSS_Rat_Triobp VSQNT----------------------------------------------PRTSSTQVD 519

. *:. **:**** *

Human_TRIOBP NPKTSCTKRDNLRPTCTQRDRTQSFSFQRDNPGTSSSQCCTQKENLRPSSPHRSTQWNNP 892

DSS_Rat_Triobp KTTASCSRWEHLRSACTQRDNPRT-----------LSQGCTQKDNPGPSSPHRATQGSSS 568

: .:**:: ::** :*****. :: ** ****:* ******:** ..

Human_TRIOBP RNSSPHRTNKDIPWASFPLRPTQSDGPRTSSPSRSKQSEVPWASIALRPTQGDRPQTSSP 952

DSS_Rat_Triobp RNPSPHRTNKDIPWASFPLRPTQSDSSRTSSPSRTKQNQVPWASISLRPTQGDKPQTSAP 628

** **********************. *******:**.:******:*******:****:*

Human_TRIOBP SRPAQHDPPQSSFGPTQYNLPSRATSSSHNPGHQSTSRTSSPVYPAAYGAPLTSPEPSQP 1012

DSS_Rat_Triobp SKLAHNDPPQQYS-PS----LATSSSSSHNPGHPSASRTSSPLHPAPRGAPQTSLEPSQP 683

*: *::****. *: : ::******** *:******::** *** ** *****

Human_TRIOBP PCAVCIGHRDAPRASSPPRYLQHDPFPFFPEPRAPESEPPHHEPPYIPPAVCIGHRDAPR 1072

DSS_Rat_Triobp PCAVCIGHRDAPRASSPPRYFQYDPFPFFPDPRSSESESPHHEPPYMPPAVCIGHRDAPR 743

********************:*:*******:**: *** *******:*************

Human_TRIOBP ASSPPRHTQFDPFPFLPDTSDAEHQCQSPQHEPLQLPAPVCIGYRDAPRASSPPRQAPEP 1132

DSS_Rat_Triobp ATSPPRHTQFDPFPFLPDTSDAEN--ESPQHDPPQFPPPVCIGYRDAPRASSPPRQFPEP 801

*:*********************: :****:* *:* ****************** ***

Human_TRIOBP SLLFQDLPRASTESLVPSMDSLHECPHIPTPVCIGHRDAPSFSSPPRQAPEPSLFFQDPP 1192

DSS_Rat_Triobp S-FFQDLPRASTESLVPSTDSMHEPPHIPTPVCIGHRDAPSFSSPPRQAPEPSLFFQDPP 860

* :*************** **:** ***********************************

Human_TRIOBP GTSMESLAPSTDSLHGSPVLIPQVCIGHRDAPRASSPPRHPPSDLAFLAPSPSPGSSGGS 1252

DSS_Rat_Triobp GTSMESLAPSVDSLHGSPLLLPQVCIGHRDAPRASSPPRHPPSDVGILAPSPPPGSS-GS 919

**********.*******:*:***********************:.:***** **** **

Human_TRIOBP RGSAPPGETRHNLEREEYTVLADLPPPRRLAQRQPGPQAQCSSGGRTHSPGRAEVERLFG 1312

DSS_Rat_Triobp RGSAPPGETRHNLEREEYTMLADLPPPRRLAQRVPEPQAQGSNEGRTRSPGRAEVERLFG 979

*******************:************* * **** *. ***:************

Human_TRIOBP QERRKSEAAGAFQAQDEGRSQQPSQGQSQLLRRQSSPAPSRQVTMLPAKQAELTRRSQAE 1372

DSS_Rat_Triobp QERRKSEAPGAFQARDEGRSQRPSQGQSQL-RRQSSPAPSRQVTKPSAKQAEPTRQSRTG 1038

******** *****:******:******** ************* ***** **:*::

Human_TRIOBP PPHPWSPEKRPEGDRQLQGSPLPPRTSARTPERELRTQRPLESGQAGPRQPLGVWQSQEE 1432

DSS_Rat_Triobp PPHPKSPEKHSEGDRQLQRIAPPARTSARPPERKAQIERHLESGYTGLRQPLGGWQSREG 1098

**** ****: ******* * ***** ***: : :* **** :* ***** ***:*

Human_TRIOBP PPGSQGPHRHLERSWSSQEGGLGPGGWWGCGEPSLGAAKAPEGAWGGTSREYKESWGQPE 1492

DSS_Rat_Triobp LSGPQSPNRHPEKNWGSQEEGLSLGGWPELGGPSL------EGIWRGPPQEHRERWGQSE 1152

* *.*:** *:.*.*** **. *** * *** ** * * :*::* *** *

Human_TRIOBP AWEEKPTHELPRE----LG-------KRSPLTSPPENWGGPAESSQSWHSGTPTAVGWGA 1541

DSS_Rat_Triobp AWEEPPSNGIQGGPPRGQGSLQELPRPHQPPPSPENSWAGPAECFCARQPEAGTAMGWRA 1212

**** *:: : * :.* ** :.*.****. : : : **:** *

Human_TRIOBP EGACPYPRGSERRPELDWRDLLGLLRAPGEGVWARVPSLDWEGLLELLQARLPRKDPAGH 1601

DSS_Rat_Triobp EGTSPHQYSAERPPDLDWRDLLGLLRAPEDGAWTRLPRLDWEGLLELLQARLPQKDPTRH 1272

**:.*: .:** *:************* :*.*:*:* ***************:***: *

Human_TRIOBP RDDLARALGPELGPPGTNDVPEQESHSQPEGWAEATPVNGHSPALQSQSPVQLPSPACTS 1661

DSS_Rat_Triobp SRDPAKAPGPEPGSSDTEDTLKTESQTQPEGWAKATLANGHRPGQQSESPAQLPSPACTS 1332

* *:* *** * .*:*. : **::******:** .*** *. **:**.*********

Human_TRIOBP TQWPKIKVTRGPATATLAGLEQTGPLGSRSTAKGPSLPELQFQPEEPEESEPSRGQDPLT 1721

DSS_Rat_Triobp TQWPTTKVTSGPETSPPVALEQIDHLES------HSPPDLEFQPEEPEASEPSRGEDSLA 1386

****. *** ** *: ..*** . * * * *:*:******* ******:* *:

Human_TRIOBP DQKQADSADKRPAEGKAGSPLKGRLVTSWRMPGDRPTLFNPFLLSLGVLRWRRPDLLNFK 1781

DSS_Rat_Triobp DQKQADSADKRPAEGKAGSPLKGRLVTSWRMPGDRPALFNPYLLSLGVLRWQRPDLLNFK 1446

************************************:****:*********:********

Human_TRIOBP KGWMSILDEPGEPPSPSLTTTSTSQWKKHWFVLTDSSLKYYRDSTAEEADELDGEIDLRS 1841

DSS_Rat_Triobp KGWMSILDEPG-------------EWKKHWFVLTDSSLKYYRDSTAEEADELDGEIDLRS 1493

*********** :***********************************

Human_TRIOBP CTDVTEYAVQRNYGFQIHTKDAVYTLSAMTSGIRRNWIEALRKTVRPTSAPDVTKLSDSN 1901

DSS_Rat_Triobp CTDVTEYAVQRNYGFQIHTKDAVYTLSAMTSGIRRNWIEALRKTVRPTSAPDVTKLSECN 1553

*********************************************************:.*

Human_TRIOBP KENALHSYSTQKGPLKAGEQRAGSEVISRGGPRKADGQRQALDYVELSPLTQASPQRART 1961

DSS_Rat_Triobp KENTLHGYGTQKSSLKIGEQRTGSEVIGRGGPRKVDGSRQSLDYVELSPLTPSSPQRVRT 1613

***:**.*.***. ** ****:*****.******.**.**:********** :****.**

Human_TRIOBP PAR-TPDRLAKQEELERDLAQRSEERRKWFEATDSRTPEVPAGEGPRRGLGAPLTEDQQN 2020

DSS_Rat_Triobp LSRSTPERPSKQEDLERDLAQRSEERRKWFESTDSRTPETPSGDGSRRGLGAPLTDDQQS 1673

:* **:* :***:*****************:*******.*:*:* *********:***.

Human_TRIOBP RLSEEIEKKWQELEKLPLRENKRVPLTALLNQSRGERRGPPSDGHEALEKEVQALRAQLE 2080

DSS_Rat_Triobp RLSEEIEKKWQELEKLPLRENKRVPLTALLNQGHSERRGPTSDSHEALEKEVQSLRAQLE 1733

********************************.:.***** **.*********:******

Human_TRIOBP AWRLQGEAPQSALRSQEDGHIPPGYISQEACERSLAEMESSHQQVMEELQRHHERELQRL 2140

DSS_Rat_Triobp AWRLRGEAPQNAPRLQEDSHIPPGYISQEACERSLAEMESSHQQVMEQLQRHHERELQRL 1793

****:*****.* * ***.****************************:************

Human_TRIOBP QQEKEWLLAEETAATASAIEAMKKAYQEELSRELSKTRSLQQGPDGLRKQHQSDVEALKR 2200

DSS_Rat_Triobp QQEKEWLLAEETAATASAIEAMKKAYQEELSRELSKTRSLQQGPDSLRKQHQLDMEALKQ 1853

*********************************************.****** *:****:

Human_TRIOBP ELQVLSEQYSQKCLEIGALMRQAEEREHTLRRCQQEGQELLRHNQELHGRLSEEIDQLRG 2260

DSS_Rat_Triobp ELQVLSERYSQKCLEIGALTRQAEEREHTLRCCQQEGQELLRHNQELHSHLSEEIDRLRS 1913

*******:*********** *********** ****************.:******:**.

Human_TRIOBP FIASQGMGNGCGRSNERSSCELEVLLRVKENELQYLKKEVQCLRDELQMMQKDKRFTSGK 2320

DSS_Rat_Triobp FIASQGTGNSCGRSNERSSCELEVLLRVKENELQYLKKEVQCLRDELQVIQKDKRFT-GK 1972

****** **.**************************************::******* **

Human_TRIOBP YQDVYVELSHIKTRSEREIEQLKEHLRLAMAALQEKESMRNSLAE 2365

DSS_Rat_Triobp YQDVYVELNHIKTRSEREIEQLKEHLRLAMAALQEKEAVRNSLAE 2017

********.****************************::******

Footnote for table: * indicates amino acid identity (73%) between human and the rat. Probable human missense mutations (The Genomes Project et al. 2015) are shaded, which were curated from <https://www.ncbi.nlm.nih.gov/snp/?term=triobp+missense> and as of July 9, 2019. Only those missense mutations with minor alleles that were observed at least 2 times (marked in red) in the tested populations are included with the validation status by 1000Genomes. Amino acids in blue indicate that the minor allele has been observed more than 10 times. The rat missense mutation has been experimentally confirmed and shaded in green. DSS, Dahl salt-sensitive rats.

**Supplemental Table 4 (E). Amino acid alignment and missense mutations in codons for trinucleotide repeat containing adaptor 6B (TNRC6B) in humans and rats**

Human_TNRC6B ------------------------------------MREKEQEREEQLMEDKKRKKEDKK 24

DSS_Rat_Tnrc6b MHTNEGEVEEESSSQVEQEDFVMEGHGKTPPPGEESKQEKEQEREEQLMEDKKRKKEDKK 60

:**********************

Human_TNRC6B KKEATQKVTEQKTKVPEVTKPSLSQPTAASPIGSSPSPPVNGGNNAKRVAVPNGQPPSAA 84

DSS_Rat_Tnrc6b KKEATQKVTEQKTKVPEVTKPSLSQPTAASPIGSSPSPPVNGGNNAKRVAVPNGQPPSAA 120

************************************************************

Human_TNRC6B RYMPREVPPRFRCQQDHKVLLKRGQPPPPSCMLLGGGAGPPPCTAPGANPNNAQVTGALL 144

DSS_Rat_Tnrc6b RYMPREVPPRFRCQQDHKVLLKRGQPPPPSCMLLGGGAGPPPCTAPGANPNNAQVTGALL 180

************************************************************

Human_TNRC6B QSESGTAPDSTLGGAAASNYANSTWGSGASSNNGTSPNPIHIWDKVIVDGSDMEEWPCIA 204

DSS_Rat_Tnrc6b QSESGTAPESTLGGAAASNYANSTWGPGASSNNGASPNPIHIWDKVIVDGSDMEEWPCIA 240

********:***************** *******:*************************

Human_TNRC6B SKDTESSSENTTDNNSASNPGSEKSTLPGSTTSNKGKGSQCQSASSGNECNLGVWKSDPK 264

DSS_Rat_Tnrc6b SKDTESSSENTTDNNSASNPGSEKSALPGSTTSNKGKGSQCQSASSGNECNLGVWKSDPK 300

*************************:**********************************

Human_TNRC6B AKSVQSSNSTTENNNGLGNWRNVSGQDRIGPGSGFSNFNPNSNPSAWPALVQEGTSRKGA 324

DSS_Rat_Tnrc6b AKSVQSPNSTSDSNNGLGTWRSTSGQDRIGPGSGFSNFNPNSNPSAWPALVQEGTCRKGV 360

****** ***::.*****.**..********************************.***.

Human_TNRC6B LETDNSNSSAQVSTVGQTSREQQSKMENAGVNFVVSGREQAQIHNTDGPKNGNTNSLNLS 384

DSS_Rat_Tnrc6b LEAEGSSSSAQVSTVGQASREQQSKMENAGVNFVVSGREQAQIHNTDGPKNGNTNSLNLS 420

**::.*.**********:******************************************

Human_TNRC6B SPNPMENKGMPFGMGLGNTSRSTDAPSQSTGDRKTGSVGSWGAARGPSGTDTVSGQSNSG 444

DSS_Rat_Tnrc6b SPNPMENKGMPFGMGLGNTSRSTDAPSQSTGDRKTGSVGSWGAARGPSGADTVSGQSNSG 480

*************************************************:**********

Human_TNRC6B NNGNNGKEREDSWKGASVQKSTGSKNDSWDNNNRSTGGSWNFGPQDSNDNKWGEGNKMTS 504

DSS_Rat_Tnrc6b NNGNNGKDREDSWKGASVPKPTGSQSDSWDNNNRSTGGSWNFGPQDSNDNKWGEGNKMTS 540

*******:********** * ***:.**********************************

Human_TNRC6B GVSQGEWKQPTGSDELKIGEWSGPNQPNSSTGAWDNQKGHPLPENQGNAQAPCWGRSSSS 564

DSS_Rat_Tnrc6b GVSQGEWKQPTGSDELKIGEWSGPNQPNSSTGAWDNQKGHPLPENQGNAQAPCWGRSSSS 600

************************************************************

Human_TNRC6B TGSEVGGQSTGSNHKAGSSDSHNSGRRSYRPTHPDCQAVLQTLLSRTDLDPRVLSNTGWG 624

DSS_Rat_Tnrc6b AGSEVGGQSTGSNHKAGSSDSHNSGRRSYRPAHPDCQAVLQTLLSRTDLDPRVLSNTGWG 660

:******************************:****************************

Human_TNRC6B QTQIKQDTVWDIEEVPRPEGKSDKGTEGWESAATQTKNSGGWGDAPSQSNQMKSGWGELS 684

DSS_Rat_Tnrc6b QTQIKQDTVWDIEEVPRPEGKSDKGTEGWESAATQTKNSGGWGDAPSQSNQMKSGWGELS 720

************************************************************

Human_TNRC6B ASTEWKDPKNTGGWNDYKNNNSSNWGGGRPDEKTPSSWNENPSKDQGWGGGRQPNQGWSS 744

DSS_Rat_Tnrc6b ASTEWKDPKSTGGWNDYKNNNSSNWGGGRADEKTPSSWNESSCKDQGWGGGRQPNQGWTS 780

*********.******************* **********. .***************:*

Human_TNRC6B GKNGWGEEVDQTKNSNWESSASKPVSGWGEGGQNEIGTWGNGGNASLASKGGWEDCKRSP 804

DSS_Rat_Tnrc6b GKNGWGEEVDQVKNNNWESSANKPVSGWGEGGQSEIGTWGNGGNTNLASKGGWEDCKRSP 840

***********.**.******.***********.**********:.**************

Human_TNRC6B AWNETGRQPNSWNKQHQQQQPPQQPPPPQPEASGSWGGPPPPPPGNVRPSNSSWSSGPQP 864

DSS_Rat_Tnrc6b AWNETGRQPNSWNKQHQQQQ--QQPPPPQPEASGSWGGPPPPPQGNVRPSNSNWSSGPQP 898

******************** ********************* ********.*******

Human_TNRC6B ATPKDEEPSGWEEPSPQSISRKMDIDDGTSAWGDPNSYNYKNVNLWDKNSQGGPAPREPN 924

DSS_Rat_Tnrc6b ATPKDDEPSGWEEPSPQSISRKMDIDDGTSAWGDPNSYNYKNVNLWDKNSQGGPAPREPN 958

*****:******************************************************

Human_TNRC6B LPTPMTSKSASVWSKSTPPAPDNGTSAWGEPNESSPGWGEMDDTGASTTGWGNTPANAPN 984

DSS_Rat_Tnrc6b LPTPMTGKSASVWSKSTPPAPDNGTSAWGEPNESSPGWGEMDDAGASATGWGNTPASAPN 1018

******.************************************:***:********.***

Human_TNRC6B AMKPNSKSMQDGWGESDGPVTGARHPSWEEEEDGGVWNTTGSQGSASSHNSASWGQGGKK 1044

DSS_Rat_Tnrc6b AMKPNSKSMQDGWGESDGPVTGARHPSWEEEDDGGVWNTAGSQGSTSSHNSASWGQGGKK 1078

*******************************:*******:*****:**************

Human_TNRC6B QMKCSLKGGNNDSWMNPLAKQFSNMGLLSQTEDNPSSKMDLSVGSLSDKKFDVDKRAMNL 1104

DSS_Rat_Tnrc6b QMKCSIKG-NNDSWMNPLAKQFSNMGLLSQTEDNPSSKMDLSV----DKKFDVDKRTMNL 1133

*****:** ********************************** *********:***

Human_TNRC6B GDFNDIMRKDRSGFRPPNSKDMGTTDSGPYFEKLTLPFSNQDGCLGDEAPCSPFSPSPSY 1164

DSS_Rat_Tnrc6b GDFNDIMRKDRPGFRPPNSKDLGTTDSGPYFEK--------------------------- 1166

*********** *********:***********

Human_TNRC6B KLSPSGSTLPNVSLGAIGTGLNPQNFAARQGGSHGLFGNSTAQSRGLHTPVQPLNSSPSL 1224

DSS_Rat_Tnrc6b ------------------------------GGSHGLFGNSTAQSRGLHTPVQPLSSSPGL 1196

************************.***.*

Human_TNRC6B RAQVPPQFISPQVSASMLKQFPNSGLSPGLFNVGPQLSPQQIAMLSQLPQIPQFQLACQL 1284

DSS_Rat_Tnrc6b RAQVPPQFISPQVSASMLKQFPNSGLNPGLFNVGPQLSPQQIAMLSQLPQIPQFQLACQL 1256

**************************.*********************************

Human_TNRC6B LLQQQQQQQ-LLQNQRKISQAVRQQQEQQLARMVSALQQQQ-------------QQQQRQ 1330

DSS_Rat_Tnrc6b LLQQQQQQQQLLQNQRKISQAVRQQQEQQLARMVSALQQQQQQQQQQQQQQQQQQQQQRQ 1316

********* ******************************* ******

Human_TNRC6B PGMKHSPSHPVGPKPHLDNMVPNALNVGLPDLQTKGPIPGYGSGFSSGGMDYGMVGGKEA 1390

DSS_Rat_Tnrc6b PSMKHSPSHPVGPKPHLDNMVPNTLNVGLPDLPTKGPIPGYGSGFSSGGMDYGMVGGKEA 1376

*.*********************:******** ***************************

Human_TNRC6B GTESRFKQWTSMMEGLPSVATQEANMHKNGAIVAPGKTRGGSPYNQFDIIPGDTLGGHTG 1450

DSS_Rat_Tnrc6b GTESRFKQWTSMMEGLPSVATQEATMHKNGAIVAPGKTRGGSPYNQFDIIPGDTLGGHTG 1436

************************.***********************************

Human_TNRC6B PAGDSWLPAKSPPTNKIGSKSSNASWPPEFQPGVPWKGIQNIDPESDPYVTPGSVLGGTA 1510

DSS_Rat_Tnrc6b PAGDSWLPAKSPPTNKIGSKSSNASWPPEFQPGVPWKGIQNIDPESDPYVTPGSVLGGTA 1496

************************************************************

Human_TNRC6B TSPIVDTDHQLLRDNTTGSNSSLNTSLPSPGAWPYSASDNSFTNVHSTSAKFPDYKSTWS 1570

DSS_Rat_Tnrc6b TSPIVDTDHQLLRDNTTGSNSSLNTSLPSPGAWPYSASDNSFTNVHSTSAKFPDYKSTWS 1556

************************************************************

Human_TNRC6B PDPIGHNPTHLSNKMWKNHISSRNTTPLPRPPPGLTNPKPSSPWSSTAPRSVRGWGTQDS 1630

DSS_Rat_Tnrc6b PDPIGHNPTHLSNKMWKNHISSRNTTPLPRPPPGLTNPKPASPWSSTAPRSVRGWGTQDS 1616

****************************************:*******************

Human_TNRC6B RLASASTWSDGGSVRPSYWLVLHNLTPQIDGSTLRTICMQHGPLLTFHLNLTQGTALIRY 1690

DSS_Rat_Tnrc6b RIASASTWSDGGSVRPSYWLVLHNLTPQIDGSTLRTICMQHGPLLTFHLNLTQGTALIRY 1676

*:**********************************************************

Human_TNRC6B STKQEAAKAQTALHMCVLGNTTILAEFATDDEVSRFLAQAQPPTPAATPSAPAAGWQSLE 1750

DSS_Rat_Tnrc6b STKQEAAKAQTALHMCVLGNTTILAEFATEDEVSRFLAQAQPPTPAATPSAPATGWQSLE 1736

*****************************:***********************:******

Human_TNRC6B TGQNQSDPVGPALNLFGGSTGLGQWSSSAGGSSGADLAGASLWGPPNYSSSLWGVPTVED 1810

DSS_Rat_Tnrc6b TSQNQADPVGPALNLFGGSTGLGQWSSSAGGSSGADLAGTSLWGPPNYSSSLWGVPTVED 1796

*.***:*********************************:********************

Human_TNRC6B PHRMGSPAPLLPGDLLGGGSDSI 1833

DSS_Rat_Tnrc6b PHRMGSPAPLLPGDLLGGGSDSI 1819

***********************

Footnote for table: * indicates amino acid identity (96%) between human and the rat. Probable human missense mutations (The Genomes Project et al. 2015) are shaded, which were curated from <https://www.ncbi.nlm.nih.gov/snp/?term=tmrc6b+missense> and as of July 9, 2019. Only those missense mutations with minor alleles that were observed at least 2 times (marked in red) in the tested populations are included with the validation status by 1000Genomes. Amino acids in blue indicate that the minor allele has been observed more than 10 times. The rat missense mutation has been experimentally confirmed and shaded in green. DSS, Dahl salt-sensitive rats.

**Supplemental Table 4 (F). Amino acid alignment and missense mutations in codons for unc-51 like kinase 3 (ULK3) in humans and rats**

Human_ULK3 MAGPGWGPPRLDGFILTERLGSGTYATVYKAYAKKDTREVVAIKCVAKKSLNKASVENLL 60

DSS_Rat_Ulk3 MAGSGWGLPRLDGFILTERLGSGTYATVYKAYAKKATREVVAIKCVAKKSLNKASVENLL 60

*** *** *************************** ************************

Human_ULK3 TEIEILKGIRHPHIVQLKDFQWDSDNIYLIMEFCAGGDLSRFIHTRRILPEKVARVFMQQ 120

DSS_Rat_Ulk3 TEIEILKGIRHPHIVQLKDFQWDNDNIYLIMEFCAGGDLSRFIHTRRILPEKVARVFMQQ 120

***********************.************************************

Human_ULK3 LASALQFLHERNISHLDLKPQNILLSSLEKPHLKLADFGFAQHMSPWDEKHVLRGSPLYM 180

DSS_Rat_Ulk3 LASALQFLHERNISHLDLKPQNILLSSLEKPHLKLADFGFAQHMSPWDEKHVLRGSPLYM 180

************************************************************

Human_ULK3 APEMVCQRQYDARVDLWSMGVILYEALFGQPPFASRSFSELEEKIRSNRVIELPLRPLLS 240

DSS_Rat_Ulk3 APEMVCRRQYDARVDLWSVGVILYEALFGQPPFASRSFSELEEKIRSNRVIELPLRPQLS 240

******:***********:************************************** **

Human_ULK3 RDCRDLLQRLLERDPSRRISFQDFFAHPWVDLEHMPSGESLGRATALVVQAVKKDQEGDS 300

DSS_Rat_Ulk3 LDCRDLLQRLLERDPSRRISFQDFFAHPWVDLEHMPSGESLAQATALVVEAVKKDQEGDA 300

***************:************************.:******:*********:

Human_ULK3 AAALSLYCKALDFFVPALHYEVDAQRKEAIKAKVGQYVSRAEELKAIVSSSNQALLRQGT 360

DSS_Rat_Ulk3 AAALSLYCKALDFFVPALHYEVDAQRKEAIKAKVGQYVSRAEELKAIVSSSNQALLRQGT 360

************************************************************

Human_ULK3 SARDLLREMARDKPRLLAALEVASAAMAKEEAAGGEQDALDLYQHSLGELLLLLAAEPPG 420

DSS_Rat_Ulk3 TGQELLREMARDKPRLLAALEVASAAMAKEEEAGKEQDALDLYQHSLGELLLLLAAEAPG 420

:.::*************************** ** ********************** **

Human_ULK3 RRRELLHTEVQNLMARAEYLKEQVKMRESRWEADTLDKEGLSESVRSSCTLQ 472

DSS_Rat_Ulk3 RRRELLHTEVQNLMARAEYLKEQIKIRESHWEAESLDKEGLSESVRSSCTLQ 472

***********************:*:***:***::*****************

Footnote for table: * indicates amino acid identity (96%) between human and the rat. Probable human missense mutations (The Genomes Project et al. 2015) are shaded, which were curated from <https://www.ncbi.nlm.nih.gov/snp/?term=ulk3+missense> and as of July 9, 2019. Only those missense mutations with minor alleles that were observed at least 2 times (marked in red) in the tested populations are included with the validation status by 1000Genomes. Amino acids in blue indicate that the minor allele has been observed more than 10 times. The rat missense mutation has been experimentally confirmed and shaded in green. DSS, Dahl salt-sensitive rats.

**Supplemental Table 4 (G). Amino acid alignment and missense mutations in codons for cytochrome P450 family 1 subfamily A member 2 (CYP1A2) in humans and rats**

Human_CYP1A2 MALSQSVPFSATELLLASAIFCLVFWVLKGLRPRVPKGLKSPPEPWGWPLLGHVLTLGKN 60

DSS_Rat_Cyp1a2 MAFSQYIS-LAPELLLATAIFCLVFWVLRGTRTQVPKGLKSPPGPWGLPFIGHMLTLGKN 59

**:** : * *****:**********:* * :********* *** *::**:******

Human_CYP1A2 PHLALSRMSQRYGDVLQIRIGSTPVLVLSRLDTIRQALVRQGDDFKGRPDLYTSTLITDG 120

DSS_Rat_Cyp1a2 PHLSLTKLSQQYGDVLQIRIGSTPVVVLSGLNTIKQALVKQGDDFKGRPDLYSFTLITNG 119

***:*:::**:**************:*** *:**:****:************: ****:*

Human_CYP1A2 QSLTFSTDSGPVWAARRRLAQNALNTFSIASDPASSSSCYLEEHVSKEAKALISRLQELM 180

DSS_Rat_Cyp1a2 KSMTFNPDSGPVWAARRHLAQDALKSFSIASDPTSVSSCYLEEHVSKEANHLISKFQKLM 179

:*:**. ********** ***:**::*******:* *************: ***::*:**

Human_CYP1A2 AGPGHFDPYNQVVVSVANVIGAMCFGQHFPESSDEMLSLVKNTHEFVETASSGNPLDFFP 240

DSS_Rat_Cyp1a2 AEVGHFEPVNQVVESVANVIGAMCFGKNFPRKSEEMLNLVKSSKDFVENVTSGNAVDFFP 239

* ***:* **** ************::**..*:***.***.:::***..:*** :****

Human_CYP1A2 ILRYLPNPALQRFKAFNQRFLWFLQKTVQEHYQDFDKNSVRDITGALFKHSKKGPRASGN 300

DSS_Rat_Cyp1a2 VLRYLPNPALKRFKNFNDNFVLFLQKTVQEHYQDFNKNSIQDITGALFKHSEN-YKDNGG 298

:*********:*** **:.*: *************:***::**********:: : .*.

Human_CYP1A2 LIPQEKIVNLVNDIFGAGFDTVTTAISWSLMYLVTKPEIQRKIQKELDTVIGRERRPRLS 360

DSS_Rat_Cyp1a2 LIPQEKIVNIVNDIFGAGFETVTTAIFWSILLLVTEPKVQRKIHEELDTVIGRDRQPRLS 358

*********:*********:****** **:: ***:*::****::********:*:****

Human_CYP1A2 DRPQLPYLEAFILETFRHSSFLPFTIPHSTTRDTTLNGFYIPKKCCVFVNQWQVNHDPEL 420

DSS_Rat_Cyp1a2 DRPQLPYLEAFILEIYRYTSFVPFTIPHSTTRDTSLNGFHIPKERCIFINQWQVNHDEKQ 418

************** :*::**:************:****:***: *:*:******** :

Human_CYP1A2 WEDPSEFRPERFLTADGTAINKPLSEKMMLFGMGKRRCIGEVLAKWEIFLFLAILLQQLE 480

DSS_Rat_Cyp1a2 WKDPFVFRPERFLTNDNTAIDKTLSEKVMLFGLGKRRCIGEIPAKWEVFLFLAILLHQLE 478

*:** ******** *.***:* ****:****:********: ****:********:***

Human_CYP1A2 FSVPPGVKVDLTPIYGLTMKHARCEHVQARLRFSIN 516

DSS_Rat_Cyp1a2 FTVPPGVKVDLTPSYGLTMKPRTCEHVQAWPRFSK- 513

*:*********** ****** ****** ***

Footnote for table: * indicates amino acid identity (76%) between human and the rat. Probable human missense mutations (The Genomes Project et al. 2015) are shaded, which were curated from <https://www.ncbi.nlm.nih.gov/snp/?term=cyp1a2+missense> and as of July 9, 2019. Only those missense mutations with minor alleles that were observed at least 2 times (marked in red) in the tested populations are included with the validation status by 1000Genomes. Amino acids in blue indicate that the minor allele has been observed more than 10 times. The rat missense mutation has been experimentally confirmed and shaded in green. DSS, Dahl salt-sensitive rats.

**Supplemental Table 4 (H). Amino acid alignment and missense mutations in codons for lectin, mannose binding 1 like (LMAN1l) in humans and rats**

Human_LMAN1L MPAVSGPGPLFCLLLLLLDPHSPETGC-PPLRRFEYKLSFKGPRLALPGAGIPFWSHHGD 59

DSS_Rat_Lman1l MLKTGGLSPSLCLLSLLLALHGAERSYPPPQRRFEYKLSFKGPRLAVPGAGIPFWSHHGD 60

* ..* .* :*** *** * * . ** ***************:*************

Human_LMAN1L AILGLEEVRLTPSMRNRSGAVWSRASVPFSAWEVEVQMRVTGLGRRGAQGMAVWYTRGRG 119

DSS_Rat_Lman1l AIPGLEEVRLVPSMKNRSGAVWSEISVSFPSWEVEMQMRVTGPGRRGALGVAMWYTKDRD 120

** *******.***:********. ** * :****:****** ***** *:*:***:.*.

Human_LMAN1L HVGSVLGGLASWDGIGIFFDSPAEDTQDSPAIRVLASDGHIPSEQPGDGASQGLGSCHWD 179

DSS_Rat_Lman1l QVGSVVEGLASWDGIGIYFDSSSNDVQNGPAIRVLASDGHDLQEQFGDGTVRELGSCLRD 180

:****: **********:*** ::*.*:.*********** .** ***: : **** *

Human_LMAN1L FRNRPHPFRARITYWGQRLRMSLNSGLTPSDPGEFCVDVGPLLLVPGGFFGVSAATGTLA 239

DSS_Rat_Lman1l FRNRPHPFRARITYWRQRLRVSLSGGLTPNDPEEVCVDVEPLLLAPGGFFGVSAATSTLA 240

*************** ****:**..****.** *.**** ****.***********.***

Human_LMAN1L DDHDVLSFLTFSLSEPSPEVPPQPFLEMQQLRLARQLEGLWARLGLGTREDVTPKSDSEA 299

DSS_Rat_Lman1l DDHDVLSFLTFSLRDPGSEEALQPFTEKERFHLARKLEELKARLALGTREDTILPLNSKA 300

************* :*. * *** * : ::***:** * ***.******. :*:*

Human_LMAN1L QGEGERLFDLEETLGRHRRILQALRGLSKQLAQAERQWKKQLGPPGQARPDGGWALDASC 359

DSS_Rat_Lman1l QEEGERFFNLEDTLSRQSQILQALQALSRQMDQAEKQWKQQLGSVVQIRPEGGWNT---- 356

* ****:*:**:**.*: :*****:.**:*: ***:***:*** * **:***

Human_LMAN1L QIPSTPGRGGHLSMSLNKDSAKVGALLHGQWTLLQALQEMRDAAVRMAAEAQVSYLPVGI 419

DSS_Rat_Lman1l --------------------AKVSTLLYGQRTLIQALQEMREAAAQMASGAQVFYLPVGT 396

***.:**:** **:*******:**.:**: *** *****

Human_LMAN1L EHHFLELDHILGLLQEELRGPAKAAAKAPRPPGQPPRASSCLQPGIFLFYLLIQTVGFFG 479

DSS_Rat_Lman1l KHHFFELDQTLGLLQKDLRDLVKMTAKPPRPSGWLPGFSTCLRTSIFLFFLLIQTVGFFC 456

:***:***: *****::**. .* :** *** * * *:**: .****:*********

Human_LMAN1L YVHFRQELNKSLQECLSTGSLPLGPAPHTPRALGILRRQPLPASMPA 526

DSS_Rat_Lman1l YMNFRQELDKRLQEYLFTESISLQPALPIPRTIGVLRRQPVSPSMQA 503

*::*****:* *** * * *: * ** **::*:*****: ** *

Footnote for table: * indicates amino acid identity (86%) between human and the rat. Probable human missense mutations (The Genomes Project et al. 2015) are shaded, which were curated from <https://www.ncbi.nlm.nih.gov/snp/?term=lman1l+missense> and as of July 9, 2019. Only those missense mutations with minor alleles that were observed at least 2 times (marked in red) in the tested populations are included with the validation status by 1000Genomes. Amino acids in blue indicate that the minor allele has been observed more than 10 times. The rat missense mutation has been experimentally confirmed and shaded in green. DSS, Dahl salt-sensitive rats.

**Supplemental Table 4 (I). Amino acid alignment and missense mutations in codons for coiled-coil domain containing 33 (CCDC33) in humans and rats**

Human_CCDC33 MGLKNKKNTEDPEEPLIASQSTEPEIGHLSPSKKETIMVTLHGATNLPACKDGSEPWPYV 60

DSS_Rat_Ccdc33 MGRQKTKVPEEPQDRLDTSLDPYPDTNYLAPCNKETVMVTLYGATNLPTCKDSSEPWPYV 60

** ::.* *:*:: * :* . *: .:*:*.:***:****:******:***.*******

Human_CCDC33 VVKSTSEEKNNQSSKAVTSVTSEPTRAPIWGDTVNVEIQAEDAGQEDVILKVVDNRKKQE 120

DSS_Rat_Ccdc33 VVKTTSEEANNHSPQARTSVTSEPTRAPIWGDTVNVEIQAEDTGREDVTLKVMDSNKKEE 120

***:**** **:* :* *************************:*:*** ***:*..**:*

Human_CCDC33 LLSYKIPIKYLRVFHPYHFELVKPTESGKADEATAKTQLYATVVRKSSFIPRYIGCNHMA 180

DSS_Rat_Ccdc33 LVSYEIPIKYLRAFHPYHFELKKN---EKEDEATAKTRLYATVVRKGSLLPRYIGYDHTA 177

*:**:*******.******** * * *******:********.*::***** :* *

Human_CCDC33 LEIFLRGVNEPLANNPNPIVVIARVVPNYKEFKVSQANRDLASVGLPITPLSFPIPSMMN 240

DSS_Rat_Ccdc33 LEVFLRGVNEPLVNNPSPMVVIARVVPSYTEFKARKARQDPASVGLPLTQVSFPISSPMT 237

**:*********.***.*:********.*.***. :*.:* ******:* :**** * *.

Human_CCDC33 FDVPRVSQNGCPQLSKPGGPPEQPLWNQSFLFQGRDGATSFSEDTALVLEYYSSTSMKGS 300

DSS_Rat_Ccdc33 FDVPRVSQNGCPQLSKPGGPPEQPLWNQSFLFLGRDGATSFSEDTALVLEYYPSASMKSS 297

******************************** ******************* *:***.*

Human_CCDC33 QPWTLNQPLGISVLPLKSRLYQKMLTGKGLDGLHVERLPIMDTSLKTINDEAPTVALSFQ 360

DSS_Rat_Ccdc33 EPWTLNQPLGVSVLPLKSHLYRKMLTGKGLKGLQVERLPIFDTNLKTINGEAPSVNLAFQ 357

:*********:*******:**:********.**:******:**.*****.***:* *:**

Human_CCDC33 LLSSERPENFLTPNNSKALPTLDPKILDKKLRTIQESWSKDTVSSTMDLSTSTPREAEEE 420

DSS_Rat_Ccdc33 LLSSERPENFLTTNNSKTLPTLNPKILDENLGAIRESWSVSSLDSSQEAEEL-------- 409

************ ****:****:*****::* :*:**** .::.*: : .

Human_CCDC33 PLVPEMSHDTEMNNYRRAMQKMAEDILSLRRQASILEGENRILRSRLAQQEEEEGQGKAS 480

DSS_Rat_Ccdc33 -----QPRDVEMNNYRRAMQKMAEDILALRKQANILEEENRMLRSHLTQQSIEEEQNRAE 464

:*.*****************:**:**.*** ***:***:*:**. ** *.:*.

Human_CCDC33 EAQNTVSMKQKLLLSELDMKKLRDRVQHLQNELIRKNDREKELLLLYQAQQPQAALLKQY 540

DSS_Rat_Ccdc33 EENLAVSMKQRLLLNELDMKRLRDRVQHLQNELIRKNDREKELLLLYQAQQPQAAQLRRY 524

* : :*****:***.*****:********************************** *::*

Human_CCDC33 QGKLQKMKALEETVRHQEKVIEKMERVLEDRLQDRSKPPPLNRQQGKPYTGFPMLSASGL 600

DSS_Rat_Ccdc33 QDKLQKMKGLEDTVRHQEKVIEKMEQILEERLHERKEPAPSNRPQGKPIMDAFAPQASGI 584

*.******.**:*************::**:**::*.:* * ** **** . .***:

Human_CCDC33 PLGSMGENLPVELYSVLLAENAKLRTELDKNRHQQAPIILQQQALP-------------- 646

DSS_Rat_Ccdc33 PLGPAGENLAMDLYSMLLAENTRLRTELEKNRQQSAPIILQQQALPVDPGELGAGGDLAE 644

*** **** ::***:*****::*****:***:*.***********

Human_CCDC33 --------------------DLLSGTSDKFNLLAKLEHAQSRILSLESQLEDSARRWGRE 686

DSS_Rat_Ccdc33 RLQDTNGPGHSKYTETMPAQDFLGGTSDKFSLLAKLEQAQSRILSLENQLEESACHWARE 704

*:*.******.******:*********.***:** :*.**

Human_CCDC33 KQDLATRLQEQEKGFRHPSNSIIIEQPSALTHSMDLKQPSELEPLLPSSDSKLNKPLS-- 744

DSS_Rat_Ccdc33 KQNLAIRLQEQQHGFGQSPNSIIVDQPHFARSQGSTTPRQNLKD--EGYPGNIERPLQTH 762

**:** *****::** : ****::** . . . .:*: . .::::**.

Human_CCDC33 --PQKETANSQQT 755

DSS_Rat_Ccdc33 LTPGTRDIRHHLR 775

* .. . :

Footnote for table: * indicates amino acid identity (81%) between human and the rat. Probable human missense mutations (The Genomes Project et al. 2015) are shaded, which were curated from <https://www.ncbi.nlm.nih.gov/snp/?term=ccdc33+missense> and as of July 9, 2019. Only those missense mutations with minor alleles that were observed at least 2 times (marked in red) in the tested populations are included with the validation status by 1000Genomes. Amino acids in blue indicate that the minor allele has been observed more than 10 times. The rat missense mutation has been experimentally confirmed and shaded in green. DSS, Dahl salt-sensitive rats.

**Supplemental Table 4 (J). Amino acid alignment and missense mutations in codons for melatonin receptor 1B (MTNR1B) in humans and rats**

Human_MTNR1B MSENGSFANCCEAGGWAVRPGWSGAGSARPSRTPRPPWVAPALSAVLIVTTAVDVVGNLL 60

DSS_Rat_Mtnr1b MPDNSSIANCCAASGLAARPSWPGSAEAEPPETPRAPWVAPMLSTVVIVTTAVDFVGNLL 60

* :*.*:**** *.* *.**.* *:..*.* .*** ***** **:*:*******.*****

Human_MTNR1B VILSVLRNRKLRNAGNLFLVSLALADLVVAFYPYPLILVAIFYDGWALGEEHCKASAFVM 120

DSS_Rat_Mtnr1b VILSVLRNRKLRNAGNLFVVNLALADLVVALYPYPLILVAILHDGWVLGEIHCKASAFVM 120

******************:*.*********:**********::***.*** *********

Human_MTNR1B GLSVIGSVFNITAIAINRYCYICHSMAYHRIYRRWHTPLHICLIWLLTVVALLPNFFVGS 180

DSS_Rat_Mtnr1b GLSVIGSVFNITAIAINRYWCICHSATYHRACSQWHAPLYISLIWLLTLVALVPNFFVGS 180

******************* **** :*** :**:**:*.******:***:*******

Human_MTNR1B LEYDPRIYSCTFIQTASTQYTAAVVVIHFLLPIAVVSFCYLRIWVLVLQARRKAKPESRL 240

DSS_Rat_Mtnr1b LEYDPRIYSCTFIQTASTQYTMAVVAIHFLLPIAVVSFCYLRIWILVLQARRKAKAERKL 240

********************* ***.******************:********** * :*

Human_MTNR1B CLKPSDLRSFLTMFVVFVIFAICWAPLNCIGLAVAINPQEMAPQIPEGLFVTSYLLAYFN 300

DSS_Rat_Mtnr1b RLRPSDLRSFLTMFAVFVVFAICWAPLNCIGLAVAINPEAMALQIPEGLFVTSYFLAYFN 300

*:***********.***:*******************: ** ***********:*****

Human_MTNR1B SCLNAIVYGLLNQNFRREYKRILLALWNPRHCIQDASKGSHAEGLQSPAPPIIGVQH--Q 358

DSS_Rat_Mtnr1b SCLNAIVYGLLNQNFRREYKRILSALWSTGRCFHDASKCHLTEDLQGPVPPAAMATIPVQ 360

*********************** ***. :*::**** :*.**.*.** . *

Human_MTNR1B ADAL 362

DSS_Rat_Mtnr1b EGAL 364

.**

Footnote for table: * indicates amino acid identity (81%) between human and the rat. Probable human missense mutations (The Genomes Project et al. 2015) are shaded, which were curated from <https://www.ncbi.nlm.nih.gov/snp/?term=mtnr1b+missense> and as of July 9, 2019. Only those missense mutations with minor alleles that were observed at least 2 times (marked in red) in the tested populations are included with the validation status by 1000Genomes. Amino acids in blue indicate that the minor allele has been observed more than 10 times. The rat missense mutation has been experimentally confirmed and shaded in green. DSS, Dahl salt-sensitive rats.

**Supplemental Table 4 (K). Amino acid alignment and missense mutations in codons for sorting nexin 19 (SNX19) in humans and rats**

Human_SNX19 MKTETVPPFQETPAGSSCHLNNLLSSRKLMAVGVLLGWLLVIHLLVNVWLLCLLSALLVV 60

DSS_Rat_Snx19 ------------------------------------------------------------ 0

Human_SNX19 LGGWLGSSLAGVASGRLHLERFIPLATCPPCPEAERQLEREINRTIQMIIRDFVLSWYRS 120

DSS_Rat_Snx19 -----------------------------------------------MIIRDFVLSWYRS 13

*************

Human_SNX19 VSQEPAFEEEMEAAMKGLVQELRRRMSVMDSHAVAQSVLTLCGCHLQSYIQAKEATAG-K 179

DSS_Rat_Snx19 VSHEKTFEAEMEASMKGLVQELRRRMSIVDSHALTQRVLTLCGCHLQSYIQAKGATAKEQ 73

**:* :** ****:*************::****::* **************** *** :

Human_SNX19 NGPVEPSHLWEAYCRATAPHPAVHSPSAEVTYTRGVVNLLLQGLVPKPHLETRTGRHVVV 239

DSS_Rat_Snx19 SCPVEPSQLWDAYCQVTTPHPAMSCPTTEVTYARGIVNLILKELVPKPHLETRTGRHVVV 133

. *****:**:***:.*:****: .*::****:**:***:*: *****************

Human_SNX19 ELITCNVILPLISRLSDPDWIHLVLVGIFSKARDPAP------CPASAPEQPSVPTSLPL 293

DSS_Rat_Snx19 ELITCNVILPMISKLSDPDWIHLILVSIFSKYKQDAAQGTKPPSSPCVLEQPSVPTSLPL 193

********** **:*********:**.**** :: * . .. ***********

Human_SNX19 IAEVEQLPEGRA-SPVAAPVFLSYSEPEGSAGPSPEVEEGHEAVEGDLGGMCEERKVGNN 352

DSS_Rat_Snx19 MVEVESLPVGKASSPVAAPVHLASSEP----APSPEIEEGHEAVEGELPGMLEERKVGNN 249

:.***.** *:* *******.*: *** .****:*********:* ** ********

Human_SNX19 SSHFLQPNVRGPLFLCEDSELESPLSELGKETIMLMTPGSFLSDRIQDALCALESSQALE 412

DSS_Rat_Snx19 SSHFLQPDIRGPLFLCEDSELESPLSELGKETIMLMTPGNFLSHRIQDALCALDDSQALE 309

*******::******************************.***.*********:.*****

Human_SNX19 PKDGEAS---EGAEAEEGPGTETETGLPVSTLNSCPEIHIDTADKEIEQGDVTASVTALL 469

DSS_Rat_Snx19 PRDGEGSECMEGAEAEEAPGAETETGTLVSM-LNCPEIQIDPADKEAEQGDD-ASLTALL 367

*:***.* *******.**:***** ** .****:** **** **** **:****

Human_SNX19 EGPEKTCPSRPSCLEKDLTNDVSSLDPTLPPVLLSSSPPGPLSSATFSFEPLSSPDGPVI 529

DSS_Rat_Snx19 EEPEKPCLQRPSCLDKGLGSGACSLEPAVPPLSLSSSPPGPLSSATFSFESLSSPDGPVV 427

* *** * .*****:*.* ....**:*::**: ***************** ********:

Human_SNX19 IQNLRITGTITAREHSGTGFHPYTLYTVKYETALDGENSSGLQQLAYHTVNRRYREFLNL 589

DSS_Rat_Snx19 IQNLRITGTITAREHSGTGFHPYTLYTVKYETALSGENSSGLQQLAYHTVNRRYREFLNL 487

**********************************.*************************

Human_SNX19 QTRLEEKPDLRKFIKNVKGPKKLFPDLPFGNMDSDRVEARKSLLESFLKQLCAIPEIANS 649

DSS_Rat_Snx19 QTRLEEKPDLRKFIKNVKGPKKLFPDLPFGNMDSDRVEARKSLLESFLRQLCAIPEIANS 547

************************************************:***********

Human_SNX19 EEVQEFLALNTDARIAFVKKPFMVSRIDKMVVSAIVDTLKTAFPRSEPQSPTEELSEAET 709

DSS_Rat_Snx19 EEVQEFLALNTDARIAFVKKPFMVSRIDKMVVSAIVDTLKTAFPRSEPQSPTEELSEAEN 607

***********************************************************.

Human_SNX19 ESKPQTEGKKASKSRLRFSSSKISPALSVTEAQDKILYCLQEGNVESETLSMSAMESFIE 769

DSS_Rat_Snx19 ESKPQTEGKKASKSRLRFSSSKIAPALSIAEAQDKILYCLQEGNSESEVLSMSGMESFIE 667

***********************:****::************** ***.****.******

Human_SNX19 KQTKLLEMQPTKAPEKDPEQPPKGRVDSCVSDAAVPAQDPSNSDPGTETELADTALDLLL 829

DSS_Rat_Snx19 KQTKLLQMQPAEVPDKEPQQVPKESVDSGLLDKAVVSQELNKSDPGTETELADTAFDLIL 727

******:***::.*:*:*:* ** *** : * ** :*: .:*************:**:*

Human_SNX19 LLLTEQWKWLCTENMQKFLRLIFGTLVQRWLEVQVANLTSPQRWVQYLLLLQESIWPGGV 889

DSS_Rat_Snx19 LLLMEQWKWLCTENMQKFLRLVFGTLVQRWLEVQVANLTCPQRWAQYLRLLRESIWPGGV 787

*** *****************:*****************.****.*** **:********

Human_SNX19 LPKFPRPVRTQEQKLAAEKQALQSLMGVLPDLVVEILGVNKCRLSWGLVLESLQQPLINR 949

DSS_Rat_Snx19 LPKFPRPGRTQAQKAATEKQALQSLMGLLPDFLVEILGVHKCQLSWSLVLESFQQPLINR 847

******* *** ** *:**********:***::******:**:***.*****:*******

Human_SNX19 HLIYCLGDIILEFLDLSASVEESAATTSASDTPGNSKRMGVSS 992

DSS_Rat_Snx19 HLIYCLGDIILEFLDLSAAVEECAPTTSASDSPGSLKKMAVST 890

******************:***.* ******:**. *:*.**:

Footnote for table: * indicates amino acid identity (87%) between human and the rat. Probable human missense mutations (The Genomes Project et al. 2015) are shaded, which were curated from <https://www.ncbi.nlm.nih.gov/snp/?term=snx19+missense> and as of July 9, 2019. Only those missense mutations with minor alleles that were observed at least 2 times (marked in red) in the tested populations are included with the validation status by 1000Genomes. Amino acids in blue indicate that the minor allele has been observed more than 10 times. The rat missense mutation has been experimentally confirmed and shaded in green. DSS, Dahl salt-sensitive rats.
