# Supplementary material for: Conserved mammalian modularity of quantitative trait loci revealed human functional orthologs in blood pressure control
Source: PLoS One. 2020 Jul 23;15(7):e0235756. doi: 10.1371/journal.pone.0235756 (PMC7377405; doi:10.1371/journal.pone.0235756)
Supplement: Attachment — Submitted filename: responses to reviewers.docx [file pone.0235756.s006.docx]

Reviewer #1: Deng & Menard seek to publish a follow-up study on the genetic underpinnings of hypertension that builds from their recently published work (e.g., Deng & Menard, PMID: 31584514). In particular, the authors are seeking to reconcile findings of human epidemiology with genetic mechanisms of rodent models with tools that utilize a modularity (vs. an “omnigenic” regulation) approach. The manuscript demonstrates knowledge of the nuances involved in using respective hypertension models (humans vs. rats) and interpretation of the resulting data thereof. Also, they endeavor to harness scientific reductionism in their general approach (e.g., rat to human orthologs) to help open up more paths of translation towards effective blood pressure control in human subjects. See my comments below.

Response to Reviewer #1 summary comment: Your summary is to the point. We agree.

(1) General: As presented, it is not readily apparent that this manuscript is a significantly new and distinguished contribution relative to previous efforts (e.g., Deng & Menard 2019, PMID: 31584514). Although objectives/accomplishments are listed (Introduction, Lines 86-90), there needs to be a more clearly presented case for unfulfilled questions left over from previous efforts and how this study addressed them.

Response to Reviewer #1 comment (1): You are right. We have added a section in the introduction to present unfulfilled questions left over and how the current study planned to address them as follows.

Nevertheless, this initial work only covered a small section of the genome. Other rat chromosome regions that contain orthologs of human GWAS genes have not been studied.

(2) General: The authors could improve the manuscript throughout by considering and providing more of a balanced discussion of their findings in light of the literature. Also, as appropriate for scientific efforts and reports, statements of data should be limited to being supportive or suggestive of their conclusions rather than the only “right” or “valid” answer(s) vs. the broader peer-reviewed literature.

Response to Reviewer #1 comment (2): Thanks for pointing out the latest publication by Rhoden et al 2019, PMID: 30716211. In discussions, the results from this paper have been discussed to balance our results. A broader peer-reviewed literature is mostly presented in the Introduction. We have tried to tone down statements as you recommended throughout the manuscript as much as we can.

(3) General: Toggling various genes on and off or mutating them in a global manner with fortuitous consequences on blood pressure does not necessarily illuminate their individual (or interactive) roles in the grand scheme of blood pressure control or make them practical for understanding/treating hypertension. As an example, Chrm3 deletion specific to endothelial cells (a cell type central to determining vascular resistance and thus, blood pressure) does not appear to significantly alter peripheral vascular resistance, arterial tone, arterial blood pressure, or cardiac function (Rhoden et al 2019, PMID: 30716211). In tandem, this same study reported that the Chrm3 endothelium-specific knockout animal also contained reduced expression of mRNA transcripts for isoforms 1, 2, 4, & 5 (1, 3, 5 are Gq-type and 2 & 4 are Gi-type) by >40% in a vascular endothelium specific knockout of Chrm3 vs. wild-type. The potential for heteromeric interaction among isoforms as integral membrane proteins can not be excluded either. Again, no one study makes for a complete “right” answer but well-evidenced possibilities (and other layers of regulation supporting physiological function) outside of and/or complementary to the authors’ approaches and hypotheses should be considered as well.

Response to Reviewer #1 comment (3): We’d like to thank you again for pointing out the last publication on a *Chrm3* conditional knock out (Rhoden et al 2019, PMID: 30716211). Their results are consistent with those in our full-body knockout of *Chrm3* in that vasodilation mediated by its protein product is diminished, and acts independently of blood pressure control.

Despite an impaired vasodilation, blood pressure in our full-body *Chrm3* knock out was lowered, not raised (Deng et al 2018, PMID: 30354759), whereas depleting *Chrm3* from endothelial cells by Rhoden and coworkers did not change blood pressure. This paradox has created a physiological puzzle (Cowley 2018, PMID: 30354773). That means that blood pressure is not regulated by vasodilation *per se* mediated by the Chrm3-mediated pathway in the endothelial cells.

This separation suggests the following in the genetics of hypertension in either humans or animal models. Unknowing a precise mechanism in blood pressure regulation, the pivotal starting point in proving a QTL has to be its functional impact on blood pressure itself *in vivo*. Any ‘intermediate phenotype’ connected to blood pressure control cannot substitute this indispensable functional proof. This proof will then drive our mechanistic understandings of the gene, irrespective of our intuitive and known ideas on how blood pressure regulations should be mediated. This function-mechanism consideration can be extrapolated to any other quantitative and polygenic trait in addition to blood pressure, especially on GWAS.

Concerning *Chrm3* encoding M3R, it is further discussed in (Deng et al 2019, 10.1016/j.cjca.2018.12.029’). ‘The hypertension pathophysiology directed by M3R is largely dissociated from the M3R-mediated vaso-relaxation. However, the absence of M3R does not mean an endothelial dysfunction, only that the M3R-mediated pathway is inactivated. Many M3R-independent pathways in the endothelium remain active and likely important for BP regulation. Since BP decreases *in vivo* in the (full-body) *Chrm3*-nulls, even if endothelium-derived acetylcholine could contribute to endothelium-dependent dilation, it is likely to be compensated *in vivo* by other mechanisms such as improved renal and cardiac activities’.

A new section has been added in the Discussion.

(4) Although published previously, the authors should still at least define and specify animal models (DSS, biological sex, etc.) up front in the Animals section of Materials and Methods.

Response to Reviewer #1 comment (4): We agree with you. Some information as you requested has been added. Our basic animal model is inbred Dahl salt-sensitive (DSS) rats. Congenic strains were made by replacing distinct chromosome regions of DSS by those of inbred normotensive rats.

Reviewer #2: The authors have proposed an extension of their previous studies [15][16] to PLoS ONE. Since it is an extension, careful content delineation from the previous studies is expected. Unfortunately, it appears to me that the extension is weak and vague. I do not see any novel or sound insight into the study.

Response to Reviewer #2 general comment: We have delineated novelty in the revision. Our first-phase studies were limited to a few rat QTLs of human GWAS orthologs. They provided a proof of principle study. However, there are a lot more rat QTLs that correspond to additional human GWAS genes. We are reporting our studies on them here in this manuscript.

A sentence was added to the beginning of last section in Introduction as follows:

‘Nevertheless, this initial work only covered a small section of the genome. Other rat chromosome regions that contain orthologs of human GWAS genes have not been studied’. We hope now the novelty is clearer than our previous version.

1. Statistical analyses were not rigorously performed in the study.

Response to Reviewer #2 comment (1): Our statistical analyses have been reported extensively in our previous studies. We did not put them in our original submission to Plos One. Now in addressing your concerns, we have added statistics to the end of Material and Method section as follows:

Repeated measures' analysis of variance (ANOVA) followed by the Dunnett’s test was used to compare the significance in a difference or a lack of it in a blood pressure component between a congenic strain and the DSS parental strain. The Dunnett correction takes into account multiple comparisons as well as sample sizes among the comparing groups. In the analysis, a BP

component was compared on each day for the period of measurement among the strains.

Because blood pressures of rats were measured continuously for 2 weeks with one reading at every 2 minutes, the numbers given at the bottom of Fig. 1 represent only averaged values of mean arterial pressures for a strain during 2 weeks. A Dunnett value including “<” given in each comparison between congenic and DSS strains was the most conservative P value among all the days of comparisons.

In case that you may suggest a genome-wide statistics, it’s not relevant to comparing a congenic trait with the DSS parental rats.

2. There is not any new data or algorithm in the study.

Response to Reviewer #2 comment (2): Please see our responses to your comment (1) above.

3. The insights into BP are vague and speculative.

Response to Reviewer #2 comment (3): In our current study, we have provided the physiological evidence, for the first time, that certain human GWAS genes functionally affect blood pressure by rat QTL proxies. As you may know, GWAS results are probabilistic and statistical. No functional data can be obtained regarding the blood pressure physiology. The next phase of studies will be to molecularly identify the QTL/human GWAS gene that is involved in hypertension pathogenesis. Mechanistic insights will follow. We suppose what you meant by ‘vague and speculative’ is that a specific gene has not been molecularly identified to be responsible for hypertension pathogenesis. These are limitations of current studies that are recognized in Caveats and limitations section of the manuscript.

4. The writing is pretty confusing with lots of specific but unnecessary terminologies.

Response to Reviewer #2 comment (4): We attempted at minimizing specific terminologies in the revision. However, some terminologies are necessary to convey the scientific message, such as congenic strains, congenic knock in genetics, GWAS, SNPs, QTLs, and gene names CSK etc.
