# Supplementary material for: Conserved mammalian modularity of quantitative trait loci revealed human functional orthologs in blood pressure control
Source: PLoS One. 2020 Jul 23;15(7):e0235756. doi: 10.1371/journal.pone.0235756 (PMC7377405; doi:10.1371/journal.pone.0235756)
Supplement: Attachment — Submitted filename: responses to reviewers.docx [file pone.0235756.s007.docx]

Reviewer #1: Although some of my concerns have been sufficiently addressed while bringing up exciting questions for further study (regulation of endothelial-dependent vascular tone vs. regulation of systemic blood pressure), the revised presentation of the original manuscript appears to be a very modest effort. Remaining concerns are indicated below.

Response to Reviewer #1 summary comment: We’d like thank you for recognizing our responses to your previous questions. It seems that you agree with scientific merits of our current work such as a major physiological effect from each QTL and a redundancy of multiple QTLs in their effects on blood pressure, modularity conservation between humans and rats, separation of SNP markers originating from primate evolution from functional genes nearby on blood pressure .

We are addressing your logistic concerns below. Also, we have added new phrases in Introduction as ‘Here, we aimed at expanding the scope of rat QTL coverage to capture additional human GWAS genes in progressive stages, since we are a single investigator-driven lab. In the process, our new results have validated the reproducibility of our previous findings. We focused on distinct regions on DSS rat Chromosomes 7 and 8 that contain previously-unexplored rat blood pressure QTLs and human GWAS gene orthologs’. Hopefully, you are satisfied with them

Reviewer #1 General (1): There is a remaining concern that the authors don’t adequately present how the current manuscript is a new and distinguished contribution relative to previous efforts. The added statement (along with the rest of the last paragraph) in the Introduction does not add any information. The reader will like to know what a “small section of the genome” means and why the authors logically stopped there the first time around as a study for publication. Likewise, the reader will want to see examples or classifications of what “other rat chromosome regions” corresponding to human GWAS genes are left to study and why. The first summary paragraph in the Discussion is not altogether convincing of a new contribution either. These boundaries among studies and respective manuscripts need to be clear, especially in light of apparently new contributions (e.g., PMID: 31584514) since original review of the current manuscript.

Response to Reviewer #1 comment (1): We suspect that you were thinking of our current work as a partial overlap with our previous published work. You may wonder why we are submitting similar work on the genetics of other genome sections as a separate paper, instead of having all data put together as one publication. You wondered what justifications there is in presenting new data, but with similar conclusions.

You seem to think of our effort from the logistic stand point of view of GWASs, where hundreds of investigators were involved in consortia with thousands of supporting staff and a huge combined budget. That’s why these GWASs were able to study associations from the whole genomes from multiple populations simultaneously, and published them as such. For example, reference 8 was able to present data on the entire genome, instead of portions of them. In contrast to this consortium-based research, our studies are individual-investigator based with one part-time research assistant supported by a tiny private fund. You may have noticed the authorship of 2 persons on the current manuscript and our previous publication.

Faced with the reality of limited resources, we have to comprehensively study QTLs from the entire rat genome corresponding to all human GWAS genes in progressive stages, and fragmented these studies over a long period of time as resources became available. At each stage, we focused on a few QTLs at a time, produced results only on these few QTLs as our resources allowed, analyzed them and sent them for publication. Once the validity of this limited work is peer-reviewed as legitimate and sound, we moved on to the next stage, once again with our very limited resources, to other regions of QTLs, only a few QTLs this second time. The result of second-stage studies used the same method. From this new work, we reproduced previous findings in different sections of the rat genome, and confirmed our previous conclusion. Another stage of investigations was repeated, so on and so forth.

As far as we know, our research lab with only 2 persons is the only one in the world doing this kind of work presented in the current manuscript. That is why the reproducibility of our findings is crucial to make our conclusions scientifically valid, as least in our hands. Unfortunately, there is no consortium on the physiology of QTLs in animal models at the present. Most of animal genetic researchers we know have moved on to ‘greener pastures’ of human GWAS.

As you may know, the scope of Plos One is to publish valid findings in science with sound methods. In our current manuscript, we do not claim to report the first finding in biologically capturing human GWAS genes with rat orthologs, which was reported in our previous publication (PMID: 31584514). Nevertheless, our current findings on different sections of rat genomes are methodologically sound, and novel in functionally capturing new human GWAS genes, which are different from our previous published results. This reproducibility and confirmation on our previous conclusions are necessary to move the field forward. Newly-identified candidates for different GWAS genes provide new entry point for further gene-targeting studies. Novel mechanistic insights of hypertension pathogenesis will follow.

You may be aware that even GWASs on blood pressure in human populations were published by same investigators in progressive stages in the past 11 years in high-impact journals such as Nature and Nature Genetics. Each of these incremental publications increased size and ethnicity, decreased minor allele frequencies of study populations from previous one, and also addressed the replicability issue in epidemiology. In comparison, our physiological and functional studies of blood pressure QTLs do not need to increase the number of study subjects to be scientifically valid and to achieve sufficient power. This is because our hypertensive rat model and congenic knock in strains derived from it are inbred and homogeneous. They have been studied under a uniform environment. However, we do need to address the reproducibility issue. We have done so by analyzing different QTLs corresponding to different GWAS genes. As a result, our understanding of mechanisms of hypertension pathogenesis may have been improved with newly identified gene candidates responsible for different QTLs.

While we understand your comments on boundaries of results, we also like to bring your attention to our logistics and budgetary restraint of doing research in a 1-investigator-plus-one assistant lab. Just to let you know, we did not divide our results into separate publications, but rather we conducted and reported our progressive search from different stages and in an evolutionary time table. Based on our current work, we are planning further research, once again, in a small scale, in the future. We’ll present our future results as they come. We’d appreciate your evaluation when the time comes.

(2) General: Statements and supporting references need to be checked throughout the manuscript for validity. For example, there is a statement in the Introduction (Lines 65-66; “This difficulty…known to affect blood pressure) with citation of references that are approximately 7 years old now.
Response to Reviewer #1 comment (1): Yes, the reference is valid and the first GWAS reported on hypertension.

Reviewer #2: Please use the space provided to explain your answers to the questions above. You may also include additional comments for the author, including concerns about dual publication, research ethics, or publication ethics. (Please upload your review as an attachment if it exceeds 20,000 characters) (Limit 100 to 20000 Characters)

Response to Reviewer #2: There is no comments to respond to.

Reviewer #3: I congratulate Deng & Menard on their revision, and scientific efforts pertaining to the genetics of HTN. In this revision, they have addressed all comments. Their data is complex yet significant, and adds to their existing contribution to the literature. Higher resolution figures should be submitted.
Response to Reviewer #3 comment: Thank you for your appreciation. We have submitted our original figures in revision. Hopefully, the resolution is better.

Reviewer #4: Deng and Menard present results of their continued work aiming to identify functional genetic variants related to blood pressure. In this particular study, they report on 3 QTLs present in DSS rats that map to numerous GWAS human genes linked to blood pressure. The authors also make the point that non-coding SNPs in GWAS act as markers of nearby QTLs rather than being QTLs themselves. Since functional genomics research lags behind the rate of statistically-based human GWAS, this work represents important findings and considerations. However, as noted by the previous reviewers, these authors recently have published a similar paper with the same overall messages. While new QTLs are discussed in the present manuscript, the Discussion of this manuscript has a degree of overlap with the Discussion of the J Hypert 2020 article (citation #14).
Response to Reviewer #4 summary comment: We thank you for your appreciation of our science in functional genomics research. The following will answer your concerns.

Reviewer #4 comment (1). Although the authors modified the Introduction to state that the present analysis varied from the previous paper due to inclusion of other rat chromosome regions, I was left wondering if with this additional study a comprehensive set of the rat genome has now been evaluated by this group? How much more of the rat genome did the authors cover with this extension to the original work?

Response to Reviewer #4 comment (1): We suspect that you were thinking of our current work as a partial overlap with our previous published work. You may wonder why we are submitting similar work on the genetics of other genome sections as a separate paper, instead of having all data put together as one publication. You wondered what justifications there is in presenting new data, but with similar conclusions.

You seem to think of our effort from the logistic stand point of view of GWASs, where hundreds of investigators were involved in consortia with thousands of supporting staff and a huge combined budget. That’s why these GWASs were able to study associations from the whole genomes from multiple populations simultaneously, and published them as such. For example, reference 8 was able to present data on the entire genome, instead of portions of them. In contrast to this consortium-based research, our studies are individual-investigator based with one part-time research assistant supported by a tiny private fund. You may have noticed the authorship of 2 persons on the current manuscript and our previous publication.

Faced with the reality of limited resources, we have to comprehensively study QTLs from the entire rat genome corresponding to all human GWAS genes in progressive stages, and fragmented these studies over a long period of time as resources became available. At each stage, we focused on a few QTLs at a time, produced results only on these few QTLs as our resources allowed, analyzed them and sent them for publication. Once the validity of this limited work is peer-reviewed as legitimate and sound, we moved on to the next stage, once again with our very limited resources, to other regions of QTLs, only a few QTLs this second time. The result of second-stage studies used the same method. From this new work, we reproduced previous findings in different sections of the rat genome, and confirmed our previous conclusion. Another stage of investigations was repeated, so on and so forth.

As far as we know, our research lab with only 2 persons is the only one in the world doing this kind of work presented in the current manuscript. That is why the reproducibility of our findings is crucial to make our conclusions scientifically valid, as least in our hands. Unfortunately, there is no consortium on the physiology of QTLs in animal models at the present. Most of animal genetic researchers we know have moved on to ‘greener pastures’ of human GWAS.

As you may know, the scope of Plos One is to publish valid findings in science with sound methods. In our current manuscript, we do not claim to report the first finding in biologically capturing human GWAS genes with rat orthologs, which was reported in our previous publication (PMID: 31584514). Nevertheless, our current findings on different sections of rat genomes are methodologically sound, and novel in functionally capturing new human GWAS genes, which are different from our previous published results. This reproducibility and confirmation on our previous conclusions are necessary to move the field forward. Newly-identified candidates for different GWAS genes provide new entry point for further gene-targeting studies. Novel mechanistic insights of hypertension pathogenesis will follow.

You may be aware that even GWASs on blood pressure in human populations were published by same investigators in progressive stages in the past 11 years in high-impact journals such as Nature and Nature Genetics. Each of these incremental publications increased size and ethnicity, decreased minor allele frequencies of study populations from previous one, and also addressed the replicability issue in epidemiology. In comparison, our physiological and functional studies of blood pressure QTLs do not need to increase the number of study subjects to be scientifically valid and to achieve sufficient power. This is because our hypertensive rat model and congenic knock in strains derived from it are inbred and homogeneous. They have been studied under a uniform environment. However, we do need to address the reproducibility issue. We have done so by analyzing different QTLs corresponding to different GWAS genes. As a result, our understanding of mechanisms of hypertension pathogenesis may have been improved with newly identified gene candidates responsible for different QTLs.

While we understand your comments, we also like to bring your attention to our logistics and budgetary restraint of doing research in a 1-investigator-plus-one assistant lab. Just to let you know, we did not divide our results into separate publications, but rather we conducted and reported our progressive search from different stages and in an evolutionary time table. Based on our current work, we are planning further research, once again, in a small scale, in the future. We’ll present our future results as they come. We’d appreciate your evaluation when the time comes.

We have revised in the introduction to reflect my explanations to you above. ‘Here, we aimed at expanding the scope of rat QTL coverage to capture additional human GWAS genes in progressive stages, since we are a single investigator-driven lab. In the process, our new results have validated the reproducibility of our previous findings. We focused on distinct regions on DSS rat Chromosomes 7 and 8 that contain previously-unexplored rat blood pressure QTLs and human GWAS gene orthologs.

Reviewer #4 comment (2). Line 102 "They are basically the same as documented previously [15]." First, I believe the authors meant to cite reference 14 (not 15). Please verify. Second, this sentence would benefit from being rewritten. Stating that the methods were "basically the same" leaves one wondering if something was different and if so what? Please revise to provide clarity on how the methods differed, and if they did not differ, indicate that the methods were the same.
Response to Reviewer #4 comment (2): Reference 15 is our original ‘original’ work on QTL modularity. Reference 14 used the same method as reference 15. Now we have added both references to avoid confusion. We have deleted ‘basically’ in the sentence to state that our current method is the same as reported in our previous work.

Reviewer # 4 comment (3). The Discussion section is overly lengthly and has overlap with the original paper (citation 14). Particularly the material on noncoding GWAS SNPs serving as markers for nearby QTLs, a point that was discussed already at length in citation #14. In this manuscript, I very much appreciated the thoughtful Discussion of missing heritability in GWAS and the omnigenic hypothesis. The Discussion needs to be condensed and so reducing content that is similar to the previous publication would shorten the section.

Response to Reviewer #4 comment (3): You are right. We have deleted the similar discussion as we presented previously that you mentioned. We hope that the revised discussion is acceptable to you.
