# Supplementary material for: Conserved mammalian modularity of quantitative trait loci revealed human functional orthologs in blood pressure control
Source: PLoS One. 2020 Jul 23;15(7):e0235756. doi: 10.1371/journal.pone.0235756 (PMC7377405; doi:10.1371/journal.pone.0235756)
Supplement: Attachment — Submitted filename: responses to reviewers.docx [file pone.0235756.s008.docx]

Reviewer #1:

General comments: In response to my primary concern, the added information provided in the authors’ responses and the statement in the Introduction (Lines 83-84; “We focused…Chromosomes 7 and 8…GWAS gene orthologs”) may be sufficient for future readers. As a note, the authors’ responses could have been much more direct while simply stating what the new objective(s) is/are and that there is no significant overlap with previous efforts. The reviewer/reader shouldn’t be left “wondering”, “seeming to think”, “hoping for” or “suspecting” anything when trying to understand a scientific study/manuscript. To the reasonable extent possible, a lucid presentation of a body of work as a scientific manuscript is an absolute requirement for publication per the responsibility of the authors. Off the record, limited laboratory staff/funding is indeed a frustrating experience and I wish the best of success for the authors in moving forward with their research. See below for a final suggestion for revising the manuscript.
Response to Reviewer #1 general comments: Thank you for your appreciation and encouragements.

(1) Introduction, Line 82: Remove “…since we are a single investigator-driven lab” from the sentence as that information is not germane to the objectives and findings of the study/manuscript.
Response to Reviewer #1 comment (1): This phrase has been removed as you asked.

Reviewer #2: Thank you for addressing the comments.i don't have any comment further. The submission looks acceptable.

Response to Reviewer #2 comments: Thanks.

Reviewer #3: The authors of the manuscript entitled conserved mammalian modularity of quantitative trait loci revealed human functional orthologs in blood pressure control have addressed my comments. The figures are better. I again congratulate Deng & Menard on their revision, and scientific efforts pertaining to the genetics of HTN. In this revision. Their data is complex yet significant, and adds to their existing contribution to the literature.

Response to Reviewer #3 comments: Thanks.

Reviewer #4: Thank you for the clarification regarding the approach to your investigative program. The revision to the Introduction now clearly distinguishes the purpose of the present manuscript from the previous related work, and the revision to the Discussion reduces the overlap with the previous publication (ref #14). I have only two minor requests remaining:
1. Line 373: The word "topics" should be singular, revise to "topic".

Response to Reviewer #4 comments (1): Ok.

2. Somewhere in the text I would appreciate you noting that the rat has 21 chromosomes. This will help readers understand how much of the rat genome your work has covered and what is remaining.

Response to Reviewer #4 comments (1): We have added one sentence in introduction on line 80.
